# A precision medicine classification for treatment of acute myeloid leukemia in older patients

**DOI:** 10.1186/s13045-021-01110-5

**Published:** 2021-06-23

**Authors:** Alice S. Mims, Jessica Kohlschmidt, Uma Borate, James S. Blachly, Shelley Orwick, Ann-Kathrin Eisfeld, Dimitrios Papaioannou, Deedra Nicolet, Krzysztof Mrόzek, Eytan Stein, Bhavana Bhatnagar, Richard M. Stone, Jonathan E. Kolitz, Eunice S. Wang, Bayard L. Powell, Amy Burd, Ross L. Levine, Brian J. Druker, Clara D. Bloomfield, John C. Byrd

**Affiliations:** 1 The Ohio State University Comprehensive Cancer Center, 320 West 10th Avenue, Starling Loving Hall B302, Columbus, OH 43210 USA; 2 Alliance Statistics and Data Center, The Ohio State University Comprehensive Cancer Center, Columbus, OH USA; 3 The Ohio State University Comprehensive Cancer Center, Clara D. Bloomfield Center for Leukemia Outcomes Research, Columbus, OH USA; 4 Memorial Sloan Kettering Cancer Center, New York, NY USA; 5 Dana-Farber/Partners CancerCare, Boston, MA USA; 6 Monter Cancer Center, Hofstra Northwell School of Medicine, Lake Success, NY USA; 7 Roswell Park Comprehensive Cancer Center, Buffalo, NY USA; 8 Wake Forest Baptist Comprehensive Cancer Center, Winston-Salem, NC USA; 9 The Leukemia and Lymphoma Society, White Plains, NY USA; 10 Oregon Health and Science University, Portland, OR USA; 11 The Ohio State University Comprehensive Cancer Center, 455 CCC Wiseman Hall, 400 West 12th Avenue, Columbus, OH 43210-1228 USA

**Keywords:** Acute myeloid leukemia, Mutation, Cytogenetics, Precision medicine, Outcome

## Abstract

**Background:**

Older patients (≥ 60 years) with acute myeloid leukemia (AML) often have multiple, sequentially acquired, somatic mutations that drive leukemogenesis and are associated with poor outcome. Beat AML is a Leukemia and Lymphoma Society-sponsored, multicenter umbrella study that algorithmically segregates AML patients based upon cytogenetic and dominant molecular abnormalities (variant allele frequencies (VAF) ≥ 0.2) into different cohorts to select for targeted therapies. During the conception of the Beat AML design, a historical dataset was needed to help in the design of the genomic algorithm for patient assignment and serve as the basis for the statistical design of individual genomic treatment substudies for the Beat AML study.

**Methods:**

We classified 563 newly diagnosed older AML patients treated with standard intensive chemotherapy on trials conducted by Cancer and Leukemia Group B based on the same genomic algorithm and assessed clinical outcomes.

**Results:**

Our classification identified core-binding factor and *NPM1*-mutated/*FLT3*-ITD-negative groups as having the best outcomes, with 30-day early death (ED) rates of 0 and 20%, respectively, and median overall survival (OS) of > 1 year and 3-year OS rates of ≥ 20%. All other genomic groups had ED rates of 17–42%, median OS ≤ 1 year and 3-year OS rates of ≤ 15%.

**Conclusions:**

By classifying patients through this genomic algorithm, outcomes were poor and not unexpected from a non-algorithmic, non-dominant VAF approach. The exception is 30-day ED rate typically is not available for intensive induction for individual genomic groups and therefore difficult to compare outcomes with targeted therapeutics. This Alliance data supported the use of this algorithm for patient assignment at the initiation of the Beat AML study. This outcome data was also used for statistical design for Beat AML substudies for individual genomic groups to determine goals for improvement from intensive induction and hopefully lead to more rapid approval of new therapies.

*Trial registration* ClinicalTrials.gov Identifiers: NCT00048958 (CALGB 8461), NCT00900224 (CALGB 20202), NCT00003190 (CALGB 9720), NCT00085124 (CALGB 10201), NCT00742625 (CALGB 10502), NCT01420926 (CALGB 11002), NCT00039377 (CALGB 10801), and NCT01253070 (CALGB 11001).

**Supplementary Information:**

The online version contains supplementary material available at 10.1186/s13045-021-01110-5.

## Background

Acute myeloid leukemia (AML) is not a single entity but a multitude of diseases that differ with regard to pretreatment genetic features including cytogenetics and gene mutations [1–3]. Despite this disease heterogeneity, initial AML treatment approaches have been essentially the same for the past forty years, with patients either receiving intensive induction approaches (i.e., 7 + 3) or palliative treatment including hypomethylating agents (HMA), subcutaneous cytarabine, supportive care, or hospice care. Over the past few years, numerous new agents have been added to the treatment arsenal of AML, including venetoclax combined with HMA or subcutaneous cytarabine, *IDH1*, *IDH2*, and *FLT3* inhibitors, liposomal daunorubicin/cytarabine, gemtuzumab ozogamicin, and glasdegib combined with subcutaneous cytarabine [4–13]. In the upfront setting, venetoclax, glasdegib, ivosidenib, and liposomal daunorubicin/cytarabine are approved by the Food and Drug Administration for certain older patient populations or for patients with comorbidities that prevent them from tolerating intensive induction therapy. Although these treatments lead to improved outcomes, including increased complete remission (CR) rates, disease-free survival (DFS), and overall survival (OS) for some AML patient populations, currently none of these therapies are considered curative unless the patients are able to undergo allogeneic stem cell transplantation in initial CR.

It is well known that older AML patients (aged ≥ 60 years) have worse outcomes than younger patients, but the reasons for this are not entirely clear. Some contributing factors include higher incidence of high-risk cytogenetic and molecular genetic features, secondary or therapy-related AML, and comorbidities that limit more intensive treatment approaches including allogeneic stem cell transplantation [14]. However, even among patients with favorable-risk features such as core-binding factor (CBF) or NPM1 + /FLT3-ITD- mutated AML who are able to tolerate and undergo intensive chemotherapy, older patients have worse outcomes compared with younger patients with these same genetic characteristics [15–17]. Vasu et al. showed that a 10-year DFS rate of older AML patients treated with intensive induction who were not able to receive allogeneic transplantation in first CR was 2.4% [18].

In the era of high throughput sequencing (HTS) and the availability of targeted therapies, the question remains whether an individualized treatment approach based on the results of genetic tests performed at the time of diagnosis could improve the currently poor outcomes of older AML patients. The Leukemia and Lymphoma Society (LLS) has sought to answer this question through the Beat AML Master Study. Gene mutation analysis using HTS, cytogenetic analysis, and polymerase chain reaction (PCR)-based analysis for internal tandem duplication of the *FLT3* gene (*FLT3*-ITD) are performed at the time of diagnosis in older patients with AML in a comprehensive and timely manner. Patients are then assigned to more individualized therapy based on the presence of cytogenetic and/or mutational drivers detected in the patients’ leukemic clones by inferred variant allele frequency (VAF) [19]. However, to determine whether this approach constitutes improvement upon existing standard of care, it was necessary to have a historical perspective on particular genetic groups of older AML patients and their actual outcomes. This information allowed for study planning relative to a null hypothesis for outcome expectation in specific molecular/cytogenetic groups and provide a reference for regulatory agencies when evaluating new therapies relevant to these groups.

We analyzed data from 563 older newly diagnosed de novo AML patients treated on the Cancer and Leukemia Group B (CALGB, now part of the Alliance for Clinical Trials in Oncology) trials and retrospectively assigned them to several genetic groups based on an algorithm that incorporates targetable cytogenetic abnormalities and mutational drivers with high VAF. We aimed to determine 1) whether this algorithmic approach would lead to a genetic group assignment in the majority of patients and 2) the outcomes of patients assigned to each of the genetic groups to serve as a benchmark and allow comparisons with the results of treatment with new therapeutic agents.

## Methods

### Patients, treatment, and cytogenetic studies

We analyzed 563 adults ≥ 60 years of age with newly diagnosed de novo AML (excluding acute promyelocytic leukemia) whose pretreatment bone marrow (BM) or blood samples underwent HTS analysis [20]. Patients who underwent allogeneic transplantation in first complete remission (CR) were excluded as per required for the eligibility of the CALGB/Alliance protocols. HTS analysis was not performed in all patients with CBF AML because this subtype of AML represents an already recognized, curable entity and is at the top of the LLS prioritization schema. The patients were treated on CALGB trials which included a range of time from 1984 to 2013 with all receiving standard intensive treatment (Table 1 and further details in the Additional file 1) [21–30]. As patients on the RATIFY study (CALBG 10603) were eligible only from ages 18 to 59, there are not any patients included in our analysis who received midostaurin as part of their chemotherapy regimen. All patients were considered for outcome analyses including those who suffered early death (ED), defined as death within 30 days after starting therapy irrespective of cause. Cytogenetic analyses of pretreatment BM and/or blood samples were performed by institutional CALGB/Alliance-approved laboratories. The results were confirmed by central karyotype review [31]. Patients provided written informed consent to participate in companion protocols CALGB 8461 (cytogenetic studies), CALGB 9665 (leukemia tissue bank), and CALGB 20,202 (molecular studies), which involved collection of pretreatment BM and blood samples. Treatment protocols were in accordance with the Declaration of Helsinki and approved by the institutional review boards at each center, and all patients provided written informed consent.

**Table 1:** CALGB Protocol Chemotherapy Regimens

Protocol	Induction		Maintenance
8321 (*n* = 3)	**Randomized to:** *DNR Days 1–3 (45 mg/m^2^/day < 60 years or 30 mg/m^2^/day ≥ 60 years) Ara-C Days 1–7 (100 mg/m^2^/day) **VERSUS** *DNR Days 1–3 (45 mg/m^2^/day < 60 years or 30 mg/m^2^/day ≥ 60 years) Ara-C Days 1–7 (200 mg/m^2^/day CIV)	6-TG Days 1–10 (100 mg/m^2^ PO q12 hour × 10 doses) DNR Day 57 (45 mg/m^2^/day < 60 years or 30 mg/m^2^/day ≥ 60 years) Ara-C Days 1–10, 29–38, 57–66, 84–92 (per Induction assignment BID q12 hours × 10 doses) VCR Days 29 and 84 (2 mg/m^2^ (max 2 mg)) Prednisone Days 29–33, 84–88 (40 mg/m^2^/day × 5 days)	n/a
8525 (*n* = 41)	*DNR Days 1–3 (45 mg/m^2^/day < 60 years or 30 mg/m^2^/day ≥ 60 years) Ara-C Days 1–7 (200 mg/m^2^/day CIV)	**Randomized to 4 Cycles:** Ara-C Days 1–5 (100 mg/m^2^/day CIV) **VERSUS** Days 1–5 (400 mg/m^2^/day CIV) **VERSUS** Days 1,3,5 (3gm/m^2^ every 12 h for 6 total doses)	4 Cycles: DNR Day 1 (45 mg/m^2^/day) Ara-C Days 1–5 (100 mg/m^2^ q12 hours SQ)
8721 (*n* = 1)	Ara-C Days 1,2,8,9 (3gm/m^2^ every 12 h – 8 doses total) L-asparaginase Days 2, 9 (6000 IU/m^2^) Allowed to repeat on Days 15,16 for both agents if no response	2 Cycles: Ara-C Days 1,2,8,9 (3gm/m^2^ every 12 h – 8 doses total) L-asparaginase Days 2, 9 (6000 IU/m^2^)	n/a
8821 (*n* = 1)	*DNR Days 1–3 (45 mg/m^2^/day) Ara-C Days 1–7 (200 mg/m^2^/day CIV)	**Randomized to Course 1 followed by Course 2 VERSUS Course 2 followed by Course 1:** Course 1: (1 cycle) Mitoxantrone Days 1–3 (12 mg/m^2^/day) Diaziquone Days 1–5 (28 mg/m^2^/day CIV) Course 2: (1 cycle) Etoposide Day 1 (2400 mg/m^2^ CIV) Cytoxan Days 3–6 (50 mg/kg/day)	n/a
8923 (*n* = 38)	**Randomized to:** *DNR Days 1–3 (45 mg/m^2^/day) Ara-C Days 1–7 (200 mg/m^2^/day CIV) G-CSF Starting Day 8 **VERSUS** *DNR Days 1–3 (45 mg/m^2^/day) Ara-C Days 1–7 (200 mg/m^2^/day CIV) Placebo Starting Day 8	Course 1: (up to 4 cycles) Ara-C Days 1–5 (100 mg/m^2^ CIV) Course 2: (up to 2 cycles) Ara-C Days 1–3 (500 mg/m^2^ q 12 h × 6 doses) Mitoxantrone Days 1–3 (5 mg/m^2^ × 6 doses)	n/a
9420 (*n* = 18)	**Randomized to:** *DNR Days 1–3 (dose-escalated to MTD 40 mg/m^2^/day) Etoposide Days 1–3 (dose-escalated to MTD 60 mg/m^2^/day) Ara-C Days 1–7 (100 mg/m^2^/day CIV) PSC-833 1.5gm/kg IV Days 1–3 for 2 h, followed by 10 mg/kg/day CIV for 72 h **VERSUS** *DNR Days 1–3 (dose-escalated to MTD 40 mg/m^2^/day) Etoposide Days 1–3 (dose-escalated to MTD 60 mg/m^2^/day) Ara-C Days 1–7 (100 mg/m^2^/day CIV) No PSC-833	**Randomized to 1 Cycle:** DNR Days 1–2 (30 mg/m^2^/day) Etoposide Days 1–2 (60 mg/m^2^/day) Ara-C Days 1–5 (100 mg/m^2^/day CIV) PSC-833 1.5gm/kg IV Days 1–3 for 2 h, followed by 10 mg/kg/day CIV for 72 h **VERSUS** DNR Days 1–2 (30 mg/m^2^/day) Etoposide Days 1–2 (60 mg/m^2^/day) Ara-C Days 1–5 (100 mg/m^2^/day CIV) No PSC-833 (based on initial induction assignment)	**Randomized to:** R-IL2 (0.9 × 10^6^ SQ Days 1–14, 19–28, 33–42, 47–56, 61–70, 75–90 and 12 × 10^6^ Day 15–17,29–31, 43–45, 57–59, 71–73) **VERSUS** No maintenance
9720 (*n* = 233)	**Randomized to:** *DNR Days 1–3 (40 mg/m^2^/day) Etoposide Days 1–3 (60 mg/m^2^/day) Ara-C Days 1–7 (100 mg/m^2^/day CIV) PSC-833 1.5gm/kg IV Days 1–3 for 2 h, followed by 10 mg/kg/day CIV for 72 h **VERSUS** *DNR Days 1–3 (40 mg/m^2^/day) Etoposide Days 1–3 (60 mg/m^2^/day) Ara-C Days 1–7 (100 mg/m^2^/day CIV) No PSC-833	**Randomized to 1 Cycle:** DNR Days 1–2 (30 mg/m^2^/day) Etoposide Days 1–2 (60 mg/m^2^/day) Ara-C Days 1–5 (100 mg/m^2^/day CIV) PSC-833 Days 1–3 (1.5gm/kg IV for 2 h, followed by 10 mg/kg/day CIV for 72 h) **VERSUS** DNR Days 1–2 (30 mg/m^2^/day) Etoposide Days 1–2 (60 mg/m^2^/day) Ara-C Days 1–5 (100 mg/m^2^/day CIV) No PSC-833 (based on initial induction assignment)	**Randomized to:** R-IL2 (0.9 × 10^6^ SQ Days 1–14, 19–28, 33–42, 47–56, 61–70, 75–90 and 12 × 10^6^ Day 15–17,29–31, 43–45, 57–59, 71–73) **VERSUS** No maintenance
10201 (*n* = 168)	**Randomized to:** *DNR Days 4–6 (60 mg/m^2^/day) Ara-C Days 4–10 (100 mg/m^2^/day CIV) Oblimersen Days 1–10 (7 mg/kg/day CIV) **VERSUS** *DNR Days 4–6 (60 mg/m^2^/day) Ara-C Days 4–10 (100 mg/m^2^/day CIV) No Oblimersen	**Randomized to 2 Cycles:** Ara-C Days 4–8 (2000 mg/m^2^/daily) Oblimersen Days 1–8 (7 mg/kg/day CIV) **VERSUS** Ara-C Days 4–8 (2000 mg/m^2^/daily) No Oblimersen	n/a
10502 (*n* = 35)	*DNR Days 1–3 (60 mg/m^2^/day) Ara-C Days 1–7 (100 mg/m^2^/day CIV) Bortezomib 1.3 mg/m^2^ Days 1,4,8,11	2 Cycles: Ara-C Days 1–5 (2gm/m^2^/day) Bortezomib per dose escalation Days 1,4,8,11	n/a
10801 (*n* = 13)	*DNR Days 1–3 (60 mg/m^2^/day) Ara-C Days 1–7 (200 mg/m^2^/day CIV) Dasatinib Days 8–21 (100 mg PO Daily)	4 cycles: Ara-C Days 1,3,5 (3 gm/m^2^ q12 hours < 60 years and 1gm/m^2^ q12 hours ≥ 60 years) Dasatinib Days 1–26 (100 mg PO daily)	Dasatinib 100 mg PO daily up to 12 months
11001 (*n* = 11)	*DNR Days 1–3 (60 mg/m^2^/day) Ara-C Days 1–7 (100 mg/m^2^/day CIV) Sorafenib Days 1–7 (400 mg daily)	2 Cycles: Ara-C Days 1–5 (2 gm/m^2^/day) Sorafenib Days 1–28 (400 mg PO BID)	Sorafenib 400 mg PO BID Days 1–28 up to 12 cycles

Ara-C, cytarabine; BID, twice daily; CIV, continuous intravenous infusion; DNR, daunorubicin; gm, gram; h, hour; IU, international units; kg, kilogram; m, meter; mg, milligram; MTD, maximum tolerated dose; n, number; n/a, not applicable; PO, orally; q12, every 12; SQ, subcutaneous; VCR, vincristine; 6-TG, 6-Thioguanine

^*^Reinduction therapy allowed

### Molecular analyses

Mononuclear cells were enriched through Ficoll-Hypaque gradient centrifugation and cryopreserved until use. Genomic DNA was extracted using the DNeasy Blood and Tissue Kit (QIAGEN, Hilden, Germany). The mutational status of 81 protein-coding genes was determined centrally at The Ohio State University by targeted amplicon sequencing using two different gene panels on the MiSeq platform (Illumina, San Diego, CA; see Additional file 1 for details). MuCor was used for integrative data analysis [32]. Details about the variant calling are outlined in the Additional file 1. In addition to the 81 genes assessed by HTS, testing for *CEBPA* mutations was performed as previously described, thus resulting in mutational status of 82 genes being assessed in our study [33]. Only patients with biallelic *CEBPA* mutations were considered as mutated [2]. The presence or absence of *FLT3*-ITD, as well as quantification of the *FLT3*-ITD to *FLT3* wild-type allelic ratio, was determined as previously described [34].

### Genetic algorithm/assignment

**Fig. 1:**
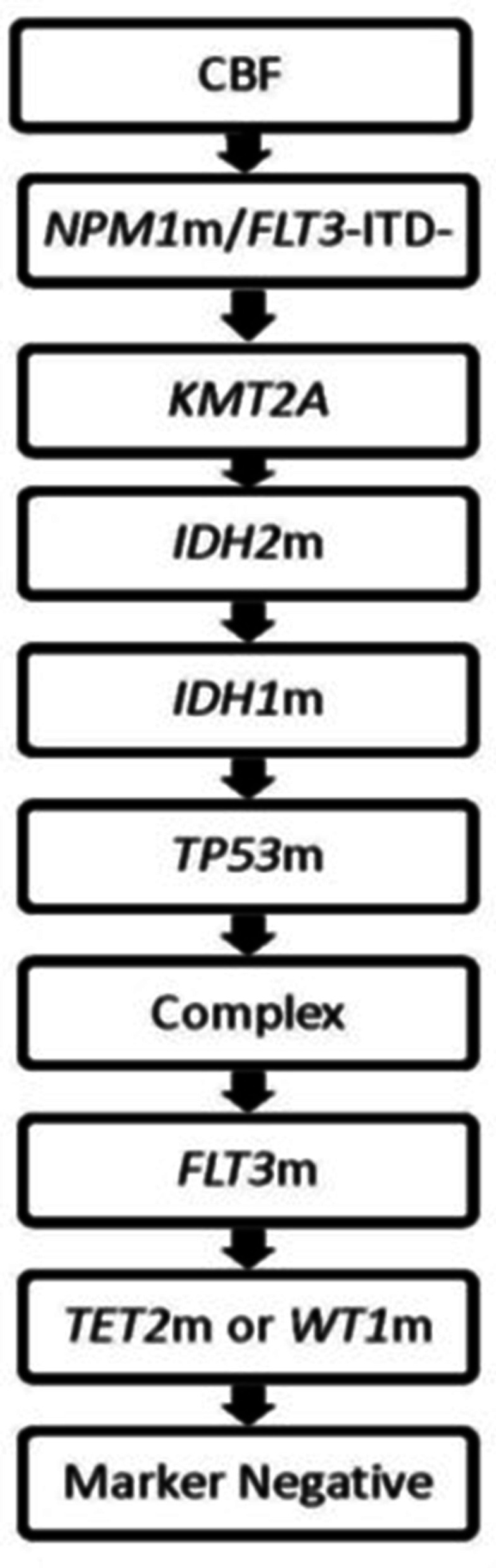
Genetic group assignment algorithm. Patients were assigned to a genetic group with initial run-through of the algorithm based on cytogenetic features or molecular mutational clones with VAF ≥ 0.3. For patients not assigned to any genetic group during the initial stratification, a second run-through of the algorithm was performed with assignment after assessing for a mutational clone with VAF ≥ 0.2. *CBF*, Core-Binding Factor; *Complex*, Complex karyotype

This precision medicine-based stratification of AML patients was initially designed in 2015 and took into consideration “assignment to curative therapy with 7 and 3” for known responsive groups [i.e., CBF AML and *NPM1-*mutated/*FLT3-*ITD-negative (*NPM1*m/*FLT3-*ITD‒) patients] followed by genetic groups where high rationale therapeutic options were or soon would be available (*KMT2A*-rearranged, *IDH2*m, *IDH1*m). These groups were first followed by high-risk genetic/cytogenetic groups which could confound prognostic impact of other gene mutations and typically lack other common co-mutations (*TP53*m and complex karyotype with wild-type *TP53*), next followed by *FLT3*-mutated [including both *FLT3*-ITD and mutations in the tyrosine kinase domain of the *FLT3* gene (*FLT3*-TKD)], then followed by the hypermethylation group [encompassing patients with *TET2*m [35, 36] or *WT1*m [37]], and then the marker-negative group. The following priority schema (in order from highest to lowest) was used for the treatment assignment algorithm: CBF AML (CBF); *NPM1*m/*FLT3-*ITD‒; 11q23/*KMT2A-*rearranged *(KMT2A*); *IDH2* mutated (*IDH2*m); *IDH1* mutated (*IDH1*m); *TP53* mutated (*TP53*m); complex karyotype/*TP53* wild-type (complex karyotype/*TP53*wt); *FLT3*-ITD (both high and low allelic ratios included) or *FLT3*-TKD (*FLT3*m); *WT1* mutated or *TET2* mutated (*WT1*m or *TET2*m); and marker-negative group (i.e., all other karyotypes and mutations that did not occur as co-mutations and were not included in the aforementioned grouping) (Fig. 1). The presence of a clonal cytogenetic aberration, *FLT3*-ITD allelic ratio of ≥ 0.05 and VAF ≥ 0.3 was assessed initially for treatment assignment. Patients were assigned in dominant clone fashion if clones harboring mutations with VAF ≥ 0.3 determined by HTS, *FLT3*-ITD allelic ratio of ≥ 0.05 or particular cytogenetic abnormalities were identified. For patients not assigned to any genetic group during the initial stratification, a second run-through of the algorithm was performed searching for a clone with mutations with VAF ≥ 0.2 excluding *FLT*3-ITD. As this algorithm is designed to assign therapy and assess outcome on an intent-to-treat basis, patients who suffered ED were included.

### Statistical analyses

We made comparisons among groups regarding baseline characteristics, co-occurring mutations, and outcomes using Fisher’s exact test for categorical variables, the Kruskal–Wallis test for continuous variables, and the Kaplan–Meier method and log rank test for survival endpoints [38]. Data collection and statistical analyses were performed by the Alliance Statistics and Data Center using SAS 9.4 and TIBCO Spotfire S + 8.2 with a dataset locked on September 12, 2019, and median follow-up of 8.6 years. Clinical endpoints are defined in the Additional file 1.

## Results

### Patient genetic group assignment

We sought to establish the outcomes of patients treated on CALGB trials who were retrospectively assigned to specific genetic groups to serve as a historical control for comparison with future results of the Beat AML trial. Using the algorithm, 498 (88%) patients were assigned to a genetic group based upon cytogenetic findings or the presence of a dominant mutational clone with VAF ≥ 0.3. This number increased to 508 (90%) when an additional 10 (2%) patients were reassigned following detection of a clone with a mutation with VAF ≥ 0.2. There were 75 (13%) patients assigned to the CBF group, 107 (19%) to the *NPM1*m/*FLT3*-ITD‒ group, 13 (2%) to the *KMT2A* group, 59 (10%) to the *IDH2*m group, 35 (6%) to the *IDH1*m group, 50 (9%) to the *TP53*m group, 28 (5%) to the complex karyotype/*TP53*wt group, 99 (18%) to the *FLT3*m group, and 42 (7%) to the *TET2*m or *WT1*m group. The remaining 56 (10%) patients were assigned to the marker-negative group (Table 2).

**Table 2:** Retrospective assignment of 589 patients receiving standard therapy on CALGB/Alliance trials to Beat AML genetic treatment groups

Assignment	Performed concurrently			Final assignment
	Step 1	Step 2	Step 3	
	Initial assignment	Initial assignment	Reassignment	
	Cytogenetics	VAF ≥ 0.3	VAF ≥ 0.2	Total number of patients *n* (%)
Core-binding factor	74	–	–	74 (13)
*NPM1*m/*FLT3-*ITD‒	–	106	1	107 (19)
*KMT2A*	13	–	–	13 (2)
*IDH2*m	–	56	3	59 (10)
*IDH1*m	–	33	2	35 (6)
*TP53*m	–	50	–	50 (9)
Complex karyotype/*TP53*wt	28	–	–	28 (5)
*FLT3*m	–	96	3	99 (18)
*TET2*m or *WT1*m	–	41	1	42 (7)
Marker-negative	–	66	− 10	56 (10)
Total number of assigned patients per column	115	448	10	563

m, mutated; n, number; VAF, variant allele frequency; wt, wild-type

### Clinical, cytogenetic and molecular genetic characteristics of patients classified into genetic groups

Baseline clinical characteristics among groups were similar with the following exceptions: (1) CBF and *NPM1*m/*FLT3-*ITD—patients had almost an equal male-to-female ratios whereas other groups had predominance of male patients; (2) platelet counts were highest in the *IDH1*m group; (3) the white blood cell counts were highest in the *FLT3*m, *KMT2A* and *NPM1*m/*FLT3-*ITD—groups; and (4) percentage of BM blasts were highest in the *KMT2A*, *IDH1*m, and the *FLT3*m groups (Table 3).

**Table 3:** Comparison of pretreatment clinical characteristics of older patients with acute myeloid leukemia assigned to the genetic groups

Characteristic	CBF *n* = 74	*NPM1*m/ *FLT3-*ITD‒ *n* = 107	*KMT2A* *n* = 13	*IDH2*m *n* = 59	*IDH1*m *n* = 35	*TP53*m *n* = 50	Complex karyotype/*TP53*wt *n* = 28	*FLT3*m *n* = 99	*TET2*m or *WT1*m *n* = 42	Marker-negative *n* = 56
Age, years										
Median Range	66 (60, 78)	68 (60, 84)	69 (60, 77)	71 (60, 86)	71 (60, 85)	70 (60, 84)	69 (61, 78)	70 (60, 89)	71 (62, 85)	69 (60, 86)
Sex, *n* (%)										
Male	37 (50)	54 (50)	7 (54)	36 (61)	19 (54)	29 (58)	20 (71)	55 (56)	26 (62)	35 (63)
Female	37 (50)	53 (50)	6 (46)	23 (39)	16 (46)	21 (42)	8 (29)	44 (44)	16 (38)	21 (38)
Race, *n* (%)										
White	61 (86)	98 (92)	11 (85)	55 (95)	31 (91)	42 (86)	23 (85)	90 (94)	38 (90)	51 (93)
Non-white	10 (14)	8 (8)	2 (15)	3 (5)	3 (9)	7 (14)	4 (15)	6 (6)	4 (10)	4 (7)
Hemoglobin, g/dL										
Median Range	8.8 (4.8, 12.4)	9.4 (5.3, 14.1)	8.5 (5.7, 11.5)	9.3 (5.2, 13.9)	9.4 (6.5, 13.8)	9.2 (6.8, 11.5)	9.4 (6.0, 14.7)	9.5 (4.3, 15.0)	9.1 (3.0, 11.9)	9.1 (6.2, 12.7)
Platelet count, × 10^9^/L										
Median Range	42 (7, 237)	75 (6, 507)	45 (8, 242)	68 (5, 673)	103 (5, 850)	44 (9, 224)	48 (4, 426)	53 (9, 387)	41 (7, 510)	56 (7, 281)
WBC count, × 10^9^/L										
Median Range	15.2 (1.4, 252.0)	29.3 (0.6, 308.5)	41.6 (2.2, 172.9)	13.4 (0.6, 434.1)	11.8 (0.7, 248.0)	7.8 (0.4, 118.0)	18.4 (0.6, 116.1)	52.4 (0.8, 450.0)	23.5 (2.2, 229.5)	14.5 (1.1, 110.0)
% Blood Blasts										
Median Range	40 (0, 96)	40 (0, 97)	71 (1, 99)	46 (0, 97)	55 (0, 99)	27 (0, 88)	34 (0, 94)	69 (0, 97)	57 (0, 99)	33 (0, 86)
% Bone marrow blasts										
Median Range	56 (10, 90)	70 (0, 95)	85 (46, 97)	70 (21, 99)	81 (34, 93)	47 (17, 90)	50 (16, 88)	80 (6, 99)	62 (26, 96)	60 (17, 94)
Extramedullary involvement, n (%)	14 (23)	28 (28)	2 (15)	15 (28)	4 (12)	7 (16)	6 (24)	28 (30)	9 (22)	7 (13)
ELN 2017, *n* (%)										
Favorable	74 (100)	107 (100)	0 (0)	6 (11)	2 (6)	1 (2)	1 (4)	22 (23)	4 (11)	4 (8)
Intermediate	0 (0)	0 (0)	8 (62)	27 (47)	16 (48)	0 (0)	0 (0)	40 (43)	16 (42)	15 (30)
Adverse	0 (0)	0 (0)	5 (38)	24 (42)	15 (45)	49 (98)	27 (96)	32 (34)	18 (47)	30 (60)
ECOG performance status, *n* (%)										
Grade 0	17 (30)	25 (26)	2 (17)	13 (23)	12 (40)	9 (20)	6 (26)	18 (20)	13 (33)	18 (38)
Grade 1	25 (44)	44 (45)	5 (42)	27 (48)	11 (37)	21 (47)	14 (61)	40 (45)	17 (43)	21 (44)
Grade 2	11 (19)	26 (27)	4 (33)	11 (20)	6 (20)	9 (20)	3 (13)	25 (28)	7 (18)	8 (17)
Grade 3	4 (7)	3 (3)	1 (8)	2 (4)	1 (3)	5 (11)	0 (0)	6 (7)	3 (8)	0 (0)
Grade 4	0 (0)	0 (0)	0 (0)	3 (5)	0 (0)	1 (2)	0 (0)	0 (0)	0 (0)	1 (2)

CBF, core-binding factor; dL, deciliter; ELN, European LeukemiaNet; g, gram; L, liter; m, mutated; n, number; WBC, white blood cell; wt, wild-type

We next analyzed occurrence of mutations belonging to the previously reported functional groups [39] within each of the genetic groups identified in the current study (Additional file 1: Table S1). We found that *NPM1*m/*FLT3-*ITD-patients had most often gene mutations in the methylation-related (87%), RAS pathway (47%), and spliceosome (28%) functional groups. In the *KMT2A* group, gene mutations were infrequent as previously reported [40] and the mutations occurring most frequently were those in genes belonging to the methylation-related (23%) functional group. Both *IDH2*m and *IDH1*m frequently had mutations in genes from spliceosome (57 and 34%, respectively), chromatin remodeling (29 and 31%), kinases (25 and 46%), and transcription factors (29 and 21%) functional groups. The most common co-mutations in *TP53*m patients were in genes from the methylation-related functional group (24%). In the complex karyotype/*TP53*wt genetic group, most often mutated were genes from spliceosome (36%) and methylation-related (32%) functional groups. The *FLT3*m genetic group had high frequency of methylation-related (59%), *NPM1* (55%), transcription factors (29%) and spliceosome (26%) mutations. Patients in the *TET2*m or *WT1*m and the marker-negative groups had high frequency of mutations in genes belonging to spliceosome (61 and 45%), transcription factors (36 and 64%), chromatin remodeling (50 and 46%), and RAS pathway (24 and 36%) functional groups (Additional file 1: Table S1).

**Fig. 2:**
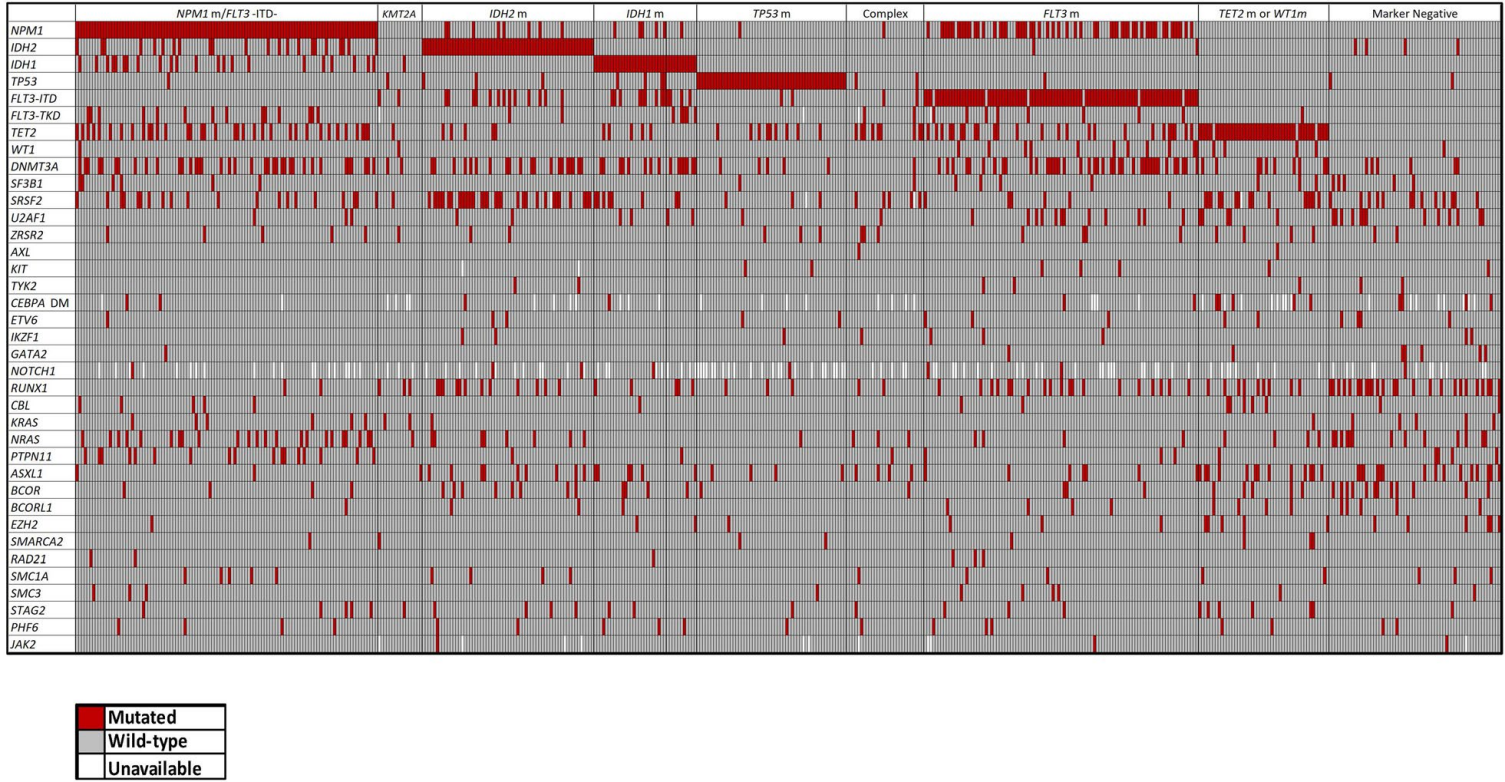
Oncoprint of co-occurring mutations found in older acute myeloid leukemia patients assigned to genetic groups. The top row colors depict each genetic group as outlined in the figure. Each column represents an individual patient and each row under the top row represents a single gene mutation. For the single gene mutation rows, the red color indicates that a gene was found to be mutated in the patient, gray indicates wild-type status of the gene, and white indicates unavailable gene mutation status. *DM*, double mutated

Frequencies of individual gene mutations in patients assigned to the genetic groups are provided in Additional file 1: Table S2. For better visualization of the mutational spectrum of each of the genetic patient groups, we created an oncoprint depicting gene mutations found in each genetic group (Fig. 2). Of note, the *NPM1*m/*FLT3-*ITD‒ genetic group had concurrently occurring mutations in the *TET2* (33%), *IDH2* (21%), and *IDH1* (21%), *TP53* (1%) genes, and *FLT3*-TKD (13%). Patients in the *IDH2*m group did not harbor a concurrent *IDH1* mutation nor did patients in the *IDH1*m group harbor simultaneously an *IDH2* mutation. The complex karyotype*/TP53*wt genetic group did include a low frequency of *TP53* mutations (*n* = 2), and in both patients the VAF of these mutations was < 0.2. The *FLT3*m genetic group had high frequency of *NPM1* (55%), *DNMT3A* (40%) and *TET2* (25%) mutations. The marker-negative genetic group included a relatively high frequency of *RUNX1* (43%), *ASXL1* (25%), *NRAS* (21%), and *U2AF1* (20%) mutations.

### Treatment outcome based on patient genetic groups

ED occurred in 20% of all patients, most commonly in the *TP53*m (42%), *KMT2A* (23%), *IDH1*m (23%), and *FLT3*m (23%) groups. All other groups had ED rates between 17 and 20%, except for the CBF group, which had no ED (Table 4). The CR rates were above 50% in the two favorable risk groups: CBF (73%), and *NPM1*m/*FLT3-*ITD‒ (68%) and 62% in the intermediate risk group KMT2A. However, in the other groups the CR rates ranged between 32 and 47%, except for *TP53*m group, in which CR rate was much lower at 16%. These CR rates were not affected by selection bias for patients surviving early AML treatment complications or progression and thus provide a historical control for new therapies in specific molecular/cytogenetic groups defined herein.

**Table 4:** Outcomes of older patients with acute myeloid leukemia assigned to the genetic groups

Outcome	CBF *n* = 74	*NPM1*m/*FLT3-*ITD- *n* = 107	*KMT2A* *n* = 13	*IDH2*m *n* = 59	*IDH1*m *n* = 35	*TP53*m *n* = 50	Complex karyotype/ *TP53*wt *n* = 28	*FLT3*m *n* = 99	*TET2*m or *WT1*m *n* = 42	Marker-negative *n* = 56
CR	55 (74)	73 (68)	8 (62)	24 (41)	14 (40)	8 (16)	9 (32)	47 (47)	16 (38)	22 (39)
Early death, *n* (%)	0 (0)	21 (20)	3 (23)	11 (19)	8 (23)	21 (42)	5 (18)	23 (23)	7 (17)	11 (20)
Death in CR, *n* (%)	6 (11)	4 (5)	3 (38)	1 (4)	1 (7)	0 (0)	0 (0)	9 (19)	0 (0)	1 (5)
Relapse rate, *n* (%)	34 (62)	59 (81)	5 (63)	23 (96)	13 (93)	8 (100)	9 (100)	37 (79)	16 (100)	18 (82)
Number expired, *n* (%)	56 (76)	95 (89)	13 (100)	59 (100)	35 (100)	50 (100)	28 (100)	97 (98)	42 (100)	52 (93)
Disease-free survival (DFS)										
Median (years) (95% CI)	0.9 (0.7–1.9)	1.0 (0.8–1.2)	0.5 (0.1–0.7)	0.9 (0.4–1.8)	0.6 (0.3–1.2)	0.4 (0.2–0.4)	0.4 (0.1–0.5)	0.5 (0.3–0.6)	0.7 (0.5–0.9)	0.7 (0.4–1.0)
% Disease-free at 1 y (95% CI)	48 (35–61)	53 (41–64)	13 (1–42)	46 (26–64)	29 (9–52)	0	11 (1–39)	15 (7–27)	13 (2–33)	41 (21–60)
% Disease-free at 3 y (95% CI)	30 (18–42)	27 (18–38)	0	8 (1–23)	0	0	0	7 (2–16)	0	14 (3–31)
% Disease-free at 5 y (95% CI)	30 (18–42)	22 (13–32)	0	4 (0–18)	0	0	0	7 (2–16)	0	14 (3–31)
Overall survival (OS)										
Median (years) (95% CI)	1.5 (1.0–2.0)	1.3 (1.0–1.5)	0.5 (0–0.8)	0.6 (0.4–0.9)	0.6 (0.2–0.8)	0.2 (0.1–0.3)	0.2 (0.1–0.5)	0.5 (0.4–0.6)	0.6 (0.4–0.9)	0.7 (0.3–1.1)
% Alive at 1 y (95% CI)	61 (48–71)	59 (49–68)	8 (0–29)	36 (24–48)	23 (11–38)	0	21 (9–38)	18 (11–26)	31 (18–45)	39 (27–52)
% Alive at 3 y (95% CI)	33 (23–44)	27 (19–36)	0	15 (8–26)	6 (1–17)	0	7 (1–20)	7 (3–13)	0	9 (3–18)
% Alive at 5 y (95% CI)	30 (20–41)	21 (14–29)	0	8 (3–17)	3 (0–13)	0	0	5 (2–11)	0	7 (2–16)

CBF, core-binding factor; CR, complete remission, m, mutated; n, number; y, year; wt, wild-type

**Fig. 3:**
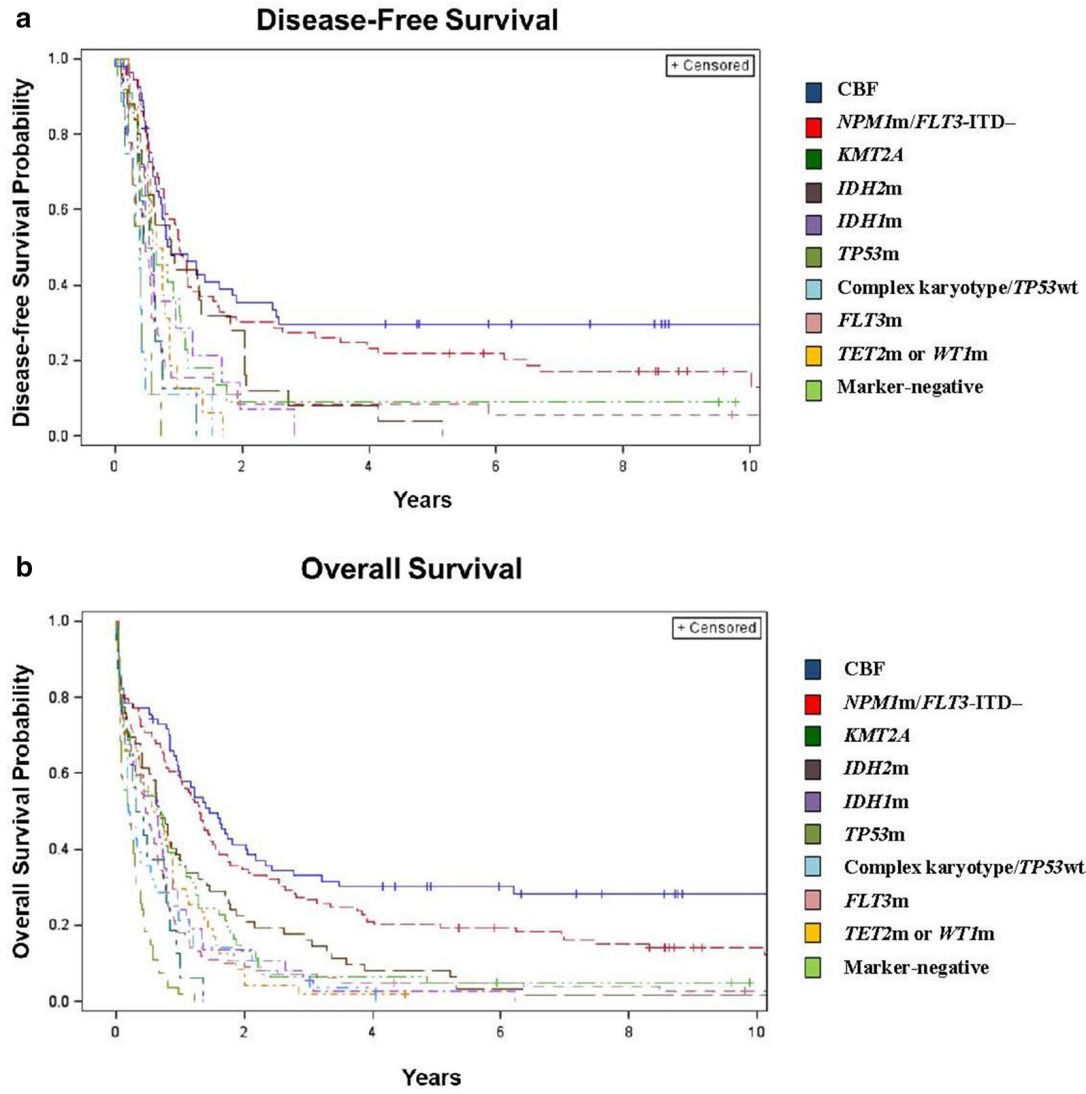
Kaplan–Meier curves depicting the **a** disease-free survival and **b** overall survival of older patients with acute myeloid leukemia classified into genetic groups. Each genetic group is identified by color as outlined in the figure

Concerning long-term outcomes, the median DFS was less than a year for all patients except for those in the *NPM1*m/*FLT3-*ITD‒ group, for whom median DFS was exactly 1 year. The 3-year DFS rates were less than 15% for all genetic groups but the two favorable groups of CBF (30%) and *NPM1*m/*FLT3-*ITD‒ (27%) that included some patients with long-term benefit from standard treatment (Table 4 and Fig. 3). Median OS was less than 1 year in all groups other than the two favorable risk ones: CBF with median OS of 1.5 years and *NPM1*m/*FLT3-*ITD‒ with median OS of 1.3 years. The 3-year OS rates were less than 10% in all groups except for CBF (33%), *NPM1*m/*FLT3-*ITD‒ (27%), and *IDH2*m (15%).

## Discussion

Our analysis demonstrates the outcomes of classifying older AML patients based on a precision-based medicine assignment of the LLS Beat AML Master Study using both targetable cytogenetic abnormalities and gene mutations found in dominant mutational clones, defined as those having VAF ≥ 0.3, or VAF ≥ 0.2 (in cases with no selected mutations with VAF ≥ 0.3), or *FLT3*-ITD allelic ratio of ≥ 0.05. The majority of patients (90%) were assigned to a genetic group as a result of the run-through of the algorithm based initially on either cytogenetic findings, *FLT3*-ITD allelic ratio of ≥ 0.05 or detection of gene mutations with a VAF ≥ 0.3, followed by a second run-through of VAF ≥ 0.2. Only 10% of patients were assigned to the marker-negative group that included patients with mutations in the spliceosome (mostly *SRSF2* and *U2AF1*), *RUNX1*, *ASXL1*, and *NRAS* genes (though the other treatment assignment genomic groups could include these mutations as well). These mutations are currently not targetable with any available therapeutic. However, as we have gained more knowledge since the original design of this algorithm, including potential mutations that lead to resistance in certain targeted therapeutics and new available therapeutics as aforementioned, other mutational genomic subgroups such as *RAS* mutated patients are being added to the algorithm and the reordering of the algorithm genomic subgroups (such as *FLT3* mutations being higher up in the stratification) are occurring. Despite this, application of this dataset did not reveal any concerns in regard to our algorithm with inappropriate genetic assignments that would preclude patients from receiving curative therapy. The outcomes of this approach prompted inclusion of this assignment algorithm in the Beat AML trial (https://www.lls.org/beat-aml).

With regard to outcomes of our patients treated with standard therapies who were assigned to genetic groups, the findings were similar to what has been reported in older AML patients. Patients in the more favorable genetic groups of CBF and *NPM1*m/*FLT3*-ITD‒ fared best, but overall the patients had poor long-term outcomes with standard treatment approaches [41, 42]. Although we are making progress with the addition of such agents as midostaurin [9], venetoclax [4], glasdegib [13, 43], and other newly approved targeted therapies to achieve short-term goals of improved CR rates, DFS and OS, these therapies remain non-curative and need to be built and improved upon further.

It is also of importance, with new treatment approaches becoming available, to understand how best to decide among multiple potential therapeutic options, both from a clinician and patient perspective. Data presented herein have served as the historical control for individual Beat AML genomic substudies statistical design at the initiation of this study and have served as benchmarks for clinical outcome improvement for particular AML genomic patient groups. This has allowed determination of clinical progress made in older AML patients and in specific genetic groups to advance novel therapies and aid in better selection of treatment. The inclusion of ED patients, who have typically been excluded from other prognostic studies, is important because this helps in assessing true outcomes of patients assigned to specific genetic groups. This information is also of value in determining if newer therapeutic options are superior to standard treatment and associated toxicities. Rates of ED observed in specific genetic groups could aid in making treatment decisions for patients that potentially influence quality of life, especially when deciding between therapies with non-curative intent. In this regard, it is notable that a very high-risk *TP53*m group had a 30-day ED rate of 41% that corresponds to the low induction success rate of 17%. These data clearly identify a distinct genetic group for which standard of care induction with 7 + 3 chemotherapy lacks therapeutic benefit. Notably, all other groups outside of CBF AML had an ED rate of 17% or more indicating that in the historical setting, 7 + 3 chemotherapy treatment and potential increased risk of infections and other complications arising from other comorbid illnesses brings early risk to elderly patients. Adaptation of functional assessment models [44, 45] or other pretreatment models [46, 47] to identify patients at risk for ED in choosing chemotherapy approaches is needed. Clinical trials in elderly AML have focused predominantly on OS as the primary endpoint for analysis, particularly when considering regulatory actions [11, 48]. Although this endpoint is of utmost importance, the potential impact of ED on the patient’s family well-being may serve to justify ED rate as another surrogate outcome for new therapies with less morbidity than chemotherapy in this disease. Examination of all aspects of ED is a major focus of the precision medicine LLS Beat AML Master Study.

The oncoprint depiction of the genetic groups defined in our prioritization is based on observations made by others [3, 49, 50]. Specifically, the *KMT2A* and *TP53*m genetic groups have very little overlap with other common AML mutations as reported by others, suggesting that both 11q23/*KMT2A* rearrangements [40] and *TP53* mutations [3] are strong drivers of the disease. Notably, no (*KMT2A*) or little (*TP53*m) overlap occurred with prognostically favorable mutations such as *NPM1* mutations or with mutations in the *IDH2* and *IDH1* genes, for which there are definitive targeted therapies with proven benefit [6–8]. As more directed FLT3 inhibitors are now available, the *FLT3*m group is moving higher up in the treatment algorithm. Also discerned from this oncoprint, one can observe that *NRAS, KRAS* and/or *PTPN11* mutations overlap with the *IDH1*m, *IDH2*m, and *FLT3*m groups but are relatively infrequent. As targeted therapies directed at *IDH2*, *IDH1*, and *FLT3* are available, it is notable that *NRAS* or *PTPN11* mutations can represent pretreatment or acquired alterations that lead to primary or secondary resistance [51, 52]. As more data comes forth from studies with targeted therapy, it has been necessary to re-examine prioritization of patients with *NRAS* and *PTPN11* mutations and potentially those with other mutations, such as *CBL* or *NF1*, which activate RAS/MAPK signaling, to include these patients as a separate genomic subgroup. Finally, examination of the marker-negative group demonstrates enrichment of patients with *RUNX1*, *ASXL1*, and spliceosome mutations that might be amendable to specific targeted therapies in the future. Future decisions to change the algorithm for the Beat AML study will also be based upon the ability to identify relevant and best directed therapeutic options.

**Fig. 4:**
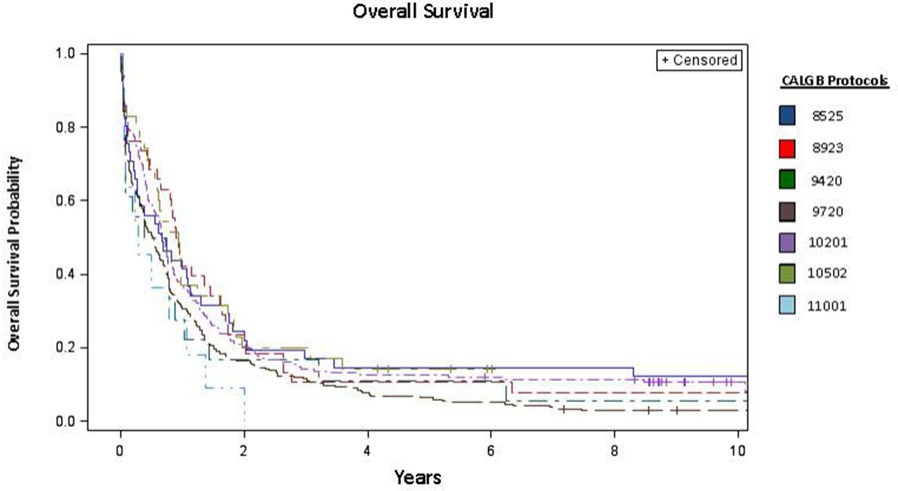
Kaplan–Meier curves depicting the overall survival of older patients with acute myeloid leukemia as treated per individual CALGB regimen. Each study is identified by color as outlined in the figure

Limitations of this study include its retrospective nature, changes in practice patterns for AML therapy over time, and lack of measurable residual disease data (MRD) to correlate with outcomes. However, despite being treated on different Alliance protocols, outcomes in regard to CR, DFS, and OS are similar between all treatment arms of the various protocols (Fig. 4). Patients received intensive regimens with different doses of anthracycline for induction and varied consolidation therapies. There was also inclusions of patients on CALGB 9720 and 9420 which were closed early due to early mortality on the investigational arms but only 32 patients in our analysis were included in treatment arms with PSC-833 with 11 of these patients being included for early death. Another major limitation is the broad range of time for the study enrollment for patients analyzed in this dataset. Supportive care for AML has improved over time with inclusion of better anti-emetics, proton pump inhibitor drugs, antifungal prophylaxis, and treatment of infectious complications and other complications, which has led to improvement in outcomes for patients and may have improvement on ED rates than what is included in our dataset. However, data from the Swedish AML Registry which began collecting patient in 2005 included newly diagnosed AML patient 60–74 with AML with *NPM1*m/*FLT3*ITD- treated with intensive chemotherapy had only slightly improved survival data compared to our findings. These patients had a median OS of 1.49 years compared to our findings of 1.33 years and 3 year OS of 35.5% in comparison to our finding of 27% [53]. Patients included in this analysis were also enrolled onto clinical trials at multiple centers, which may not be reflective of treatment given outside of clinical trials. Also our patients were not transplanted in first CR, which likely contributes to poor long-term outcomes; however, this is likely more akin to real-world data as the majority of older AML patients are, unfortunately, still not being considered for transplantation and many still remain untreated [54–56]. Albeit, this may hopefully improve with additional new less intensive treatment options that be more feasible to give in the community setting. Also, our analysis included only patients whose AML had both cytogenetic and mutational studies performed from diagnostic samples, a potential selection bias. Finally, our analysis lacks any MRD assessments and correlation to patient clinical outcomes although efforts to add this to Alliance/CALGB patient dataset analyses are currently underway. The use of MRD assessments in AML is evolving in regard to methodology and standardization, as well as timing and threshold of meaningful MRD positivity [57]. Although assessment of AML MRD remains complicated, current efforts are underway to implement multimodality MRD testing in clinical trials including the Beat AML study. It is hopeful that MRD testing can become standardized and a routine part of AML patient clinical care in order to continue to improve treatment outcomes. Despite these limitations to our dataset, our study represents one of the largest series of older AML patients with inclusion of all newly diagnosed patients regardless of early death in order to most accurately define outcomes of specific genetic groups relevant to the ongoing Beat AML study.

## Conclusions

As more treatments in AML are explored in the upfront setting, this historical outcome data from patients treated with standard treatment on CALGB/Alliance protocols can aid in determining appropriate milestone achievements for potential trial design. This dataset has been used for Beat AML genomic Phase 1/2 substudy statistical designs to determine primary endpoints. If dramatic improvements with new therapeutics are seen from these baseline expectations, the goal is to lead to rapid drug approval in this older AML population. However, as outcome data matures for newer approved treatment modalities in older AML patients, new benchmarks will be set for therapeutic clinical trials to further improve upon the recent progress that has been made in this patient population. This is particularly relevant for small genetic groups where randomized trials may not be possible. To extend the results reported herein, we will be validating our findings through analysis of a multi-institutional cohort and, ideally, in a series that includes other standards of care for elderly AML currently used in practice such as HMA [58] or venetoclax combined with HMA [4]. We also plan to assess the potential impact of co-mutations on treatment outcomes and compare data from our retrospective patient cohort in the prospective Beat AML patient data. Our hope is that genetics-based approaches will result in continued improved outcomes in both older and younger AML patient populations and lead to curative therapies, not just short-term improvements.

## Supplementary Information

**Additional file 1**. Supplementary Material.

## Data Availability

For original data, please contact Alice.Mims@osumc.edu.

## Abbreviations

AML: Acute myeloid leukemia
BM: Bone marrow
CALGB: Cancer and leukemia group B
CBF: Core-binding factor
CR: Complete remission
DFS: Disease-free survival
ED: Early death
*FLT3*m: *FLT3*-ITD (both high and low allelic ratios included) or *FLT3*-TKD
HMA: Hypomethylating agents
HTS: High throughput sequencing
*IDH1*m: *IDH1* Mutated
*IDH2*m: *IDH2* Mutated
KMT2A: 11Q23/*KMT2A-*rearranged
LLS: Leukemia and Lymphoma Society
OS: Overall survival
PCR: Polymerase chain reaction
*TET2*m: *TET2* Mutated
*TP53*m: *TP53* Mutated
*TP53*wt: *TP53* Wild-type
VAF: Variant allele frequency
*WT1*m: *WT1* Mutated
