# Supplementary material for: A precision medicine classification for treatment of acute myeloid leukemia in older patients
Source: J Hematol Oncol. 2021 Jun 23;14:96. doi: 10.1186/s13045-021-01110-5 (PMC8220739; doi:10.1186/s13045-021-01110-5)
Supplement: Supplementary file 1 — Additional file 1. Supplementary Material. [file 13045_2021_1110_MOESM1_ESM.docx]

**Supplementary Information**

**A Precision Medicine Classification for Treatment of**

**Acute Myeloid Leukemia in Older Patients**

Alice S. Mims^1^, Jessica Kohlschmidt^1,2,3^, Uma Borate^1^, James S. Blachly^1^, Shelley Orwick^1^,

Ann-Kathrin Eisfeld^1,3^, Dimitrios Papaioannou,^1^ Deedra Nicolet^1,2,3^, Krzysztof Mrόzek^1,3^,

Eytan Stein^4^, Bhavana Bhatnagar^1^, Richard M. Stone^5^, Jonathan E. Kolitz^6^, Eunice S. Wang^7^, Bayard L. Powell^8^, Amy Burd^9^, Ross L. Levine^4^, Brian J. Druker^3^, Clara D. Bloomfield^1+^, and John C. Byrd^1,3^

1. The Ohio State University Comprehensive Cancer Center, Columbus OH, USA.
2. Alliance Statistics and Data Center, The Ohio State University Comprehensive Cancer Center, Columbus, OH, USA.
3. The Ohio State University Comprehensive Cancer Center, Clara D. Bloomfield Center for Leukemia Outcomes Research, Columbus, OH, USA.
4. Memorial Sloan Kettering Cancer Center, New York, NY, USA.
5. Dana-Farber Cancer Institute, Boston, MA, USA.
6. Monter Cancer Center, Hofstra Northwell School of Medicine, Lake Success, NY, USA.
7. Roswell Park Comprehensive Cancer Center, Buffalo, NY, USA.
8. Wake Forest Baptist Comprehensive Cancer Center, Winston-Salem, NC, USA.
9. The Leukemia and Lymphoma Society.

±Deceased

**Running title:** Precision Medicine Classification of Older AML patients

**Keywords:** acute myeloid leukemia, mutation, cytogenetics, precision medicine, outcome

**Participating institutions**

The following Cancer and Leukemia Group B (CALGB)/Alliance for Clinical Trials in Oncology (Alliance) institutions participated in this study and contributed at least five patients. For each of these institutions, the current or last principal investigator and the cytogeneticists who analyzed the cases are listed as follows:

North Shore University Hospital, Manhasset, NY: Jonathan E. Kolitz, Prasad R. K. Koduru, Ayala Aviram-Goldring and Chandrika Sreekantaiah; Roswell Park Cancer Institute, Buffalo, NY: Ellis G. Levine and AnneMarie W. Block; Wake Forest University School of Medicine, Winston-Salem, NC: Heidi D. Klepin, P. Nagesh Rao, Wendy L. Flejter and Mark Pettenati; The Ohio State University Medical Center, Columbus, OH: Claire Verschraegen, Karl S. Theil, Diane Minka and Nyla A. Heerema; University of Chicago Medical Center, Chicago, IL: Hedy L. Kindler, Diane Roulston, Katrin M. Carlson, Yanming Zhang and Michelle M. LeBeau; Duke University Medical Center, Durham, NC: Jeffrey Crawford, Sandra H. Bigner, Mazin B. Qumsiyeh, John Eyre and Barbara K. Goodman; University of Iowa Hospitals, Iowa City, IA: Umar Farooq and Shivanand R. Patil; Dana Farber Cancer Institute, Boston, MA: Harold J. Burstein, Ramana V. Tantravahi, Leonard L. Atkins, Cynthia C. Morton and Paola Dal Cin; University of North Carolina, Chapel Hill, NC: Matthew I. Milowsky and Kathleen W. Rao; Washington University School of Medicine, St. Louis, MO: Nancy L. Bartlett, Michael S. Watson, Eric C. Crawford, Jaime Garcia-Heras, Peining Li and Shashikant Kulkarni; Christiana Care Health Services, Inc., Newark, DE: Gregory A. Masters, Digamber S. Borgaonkar, Jeanne M. Meck and Kathleen Richkind; Dartmouth Medical School, Lebanon, NH: Konstantin H. Dragnev, Doris H. Wurster-Hill and Thuluvancheri K. Mohandas; Rhode Island Hospital, Providence, RI: Howard P. Safran, Jennifer A. Ahearn, Hon Fong L. Mark, Shelly L. Kerman and Aurelia Meloni-Ehrig; University of Maryland Greenebaum Cancer Center, Baltimore, MD: Heather D. Mannuel, Joseph R. Testa, Maimon M. Cohen, Judith Stamberg and Yi Ning; University of Vermont Cancer Center, Burlington, VT: Peter A. Kaufman, Elizabeth F. Allen and Mary Tang; Ft. Wayne Medical Oncology/Hematology, Ft. Wayne, IN: Sreenivasa Nattam and Patricia I. Bader; Massachusetts General Hospital, Boston, MA: David Ryan, Justin Gainor, Leonard L. Atkins, Cynthia C. Morton and Paola Dal Cin; Weill Medical College of Cornell University, New York, NY: Scott T. Tagawa, Ram S. Verma, Prasad R.K. Koduru, Andrew J. Carroll and Susan Mathew; Western Pennsylvania Hospital, Pittsburgh, PA: Gene G. Finley and Gerard R. Diggans; Long Island Jewish Medical Center, Lake Success, NY: Jonathan E. Kolitz, Prasad R. K. Koduru, Ayala Aviram-Goldring and Chandrika Sreekantaiah; University of Tennessee Cancer Center, Memphis, TN: Harvey B. Niell and Sugandhi A. Tharapel; SUNY Upstate Medical University, Syracuse, NY: Stephen L. Graziano, Larry Gordon and Constance K. Stein; University of Missouri/Ellis Fischel Cancer Center, Columbia, MO: Puja Nistala, Judith H. Miles, Jeffrey R. Sawyer, Tim Hui-Ming Huang and Linda M. Pasztor; Walter Reed National Military Medical Center, Bethesda, MD: Karen G. Zeman, Rawatmal B. Surana and Nyla A. Heerema; Eastern Maine Medical Center, Bangor, ME: Sarah J. Sinclair and Laurent J. Beauregard; University of California San Diego Moores Cancer Center, San Diego, CA: Lyudmila A. Bazhenova, Renée Bernstein and Marie L. Dell'Aquila.

**Treatment protocols**

Patients in this study were treated on one of the following CALGB frontline treatment protocols: 9720 (n=233), 10201 (n=168), 8525 (n=41), 8923 (n=38), 10502 (n=35), 9420 (n=18), 10801 (n=13), 11001 (n=11), 8361 (n=3), 8821 (n=1), 8721 (n=1), 8321 (n=1).

Older patients, aged ≥60 years, enrolled onto CALGB 9720 or 9420 received induction chemotherapy consisting of cytarabine in combination with daunorubicin and etoposide, and were randomized to the arm with or without the multidrug resistance protein modulator PSC-833. The PSC-833 arm was closed after random assignment of 120 patients because of a high number of early deaths.^1-3^ Patients on CALGB 10201 received induction chemotherapy consisting of cytarabine and daunorubicin, with or without BCL2 antisense oblimersen sodium. The consolidation regimen included two cycles of cytarabine (2 g/m^2^/d) with or without oblimersen.^4^

Patients on CALGB 8525 were treated with induction chemotherapy consisting of cytarabine in combination with daunorubicin and were randomly assigned to consolidation with different doses of cytarabine followed by maintenance treatment.^5^

The patients enrolled on CALGB 8923 were treated with induction chemotherapy consisting of cytarabine in combination with daunorubicin and were randomly assigned to receive postremission therapy with cytarabine alone or in combination with mitoxantrone.^6^ Patients enrolled on CALGB 10502 received bortezomib added to both induction consisting of cytarabine and daunorubicin and to consolidation with two cycles of intermediate-dose cytarabine.^7^

CALGB 10801 enrolled patients ≥18 years old with the favorable risk, core binding factor-positive AML. Cytarabine and daunorubicin were used for remission induction, together with other agents in some studies, and a second course of induction therapy for initial nonresponders.^8^ For patients treated on CALGB 11001, sorafenib was added to the induction and consolidation treatment consisting of daunorubicin and cytarabine and consolidation with high-dose cytarabine, followed by sorafenib maintenance.^9^ CALGB 8361 is a prospective immunophenotyping study of adult AML.^10^

After induction consisting of cytarabine in combination with daunorubicin, the patients enrolled on CALGB 8821 received intensive post remission therapy with cytoxan/etoposide and diazaquone/mitoxantrone.^11^

The patient enrolled on CALGB 8721 received two courses of treatment with HiDAC plus asparaginase on days 1 and 8. CALGB 8321 patients were treated with initial induction therapy with daunorubicin and cytarabine followed by randomization to either 1 versus 2 cycles of low-dose cytarabine, daunorubicin, vincristine, and prednisone.

**Definition of clinical end points**

Complete remission (CR) required an absolute neutrophil count ≥1.5 x 10^9^/l, platelet count ≥100 x 10^9^/l, no leukemic blasts in the blood, bone marrow (BM) cellularity >20% with maturation of all cell lines, no Auer rods, ˂5% BM blast cells, and no evidence of extramedullary leukemia, all of which had persisted for at least one month. Relapse was defined by ≥5% BM blasts, circulating leukemic blasts, or the development of extramedullary leukemia. Disease-free survival was measured from the date of CR until the date of relapse or death; patients alive and relapse-free at last follow-up were censored. Overall survival was measured from the date on study until the date of death, and patients alive at last follow-up were censored.^12^

**Mutational profiling**

The mutational status of 81 protein coding genes (*AKT1*, *ARAF*, *ASXL1*, *ATM*, *AXL*, *BCL2*, *BCOR*, *BCORL1*, *BRAF*, *BRD4*, *BRINP3*, *BTK*, *CBL*, *CCND1*, *CCND2*, *CSNK1A1*, *CTNNB1*, *DNMT3A*, *ETV6*, *EZH2*, *FBXW7*, *FLT3*, *GATA1*, *GATA2*, *GSK3B*, *HIST1H1E*, *HNRNPK*, *IDH1*, *IDH2*, *IKZF1*, *IKZF3*, *ILR7*, *JAK1*, *JAK2*, *JAK3*, *KIT*, *KLHL6*, *KMT2A*, *KRAS*, *MAPK1*, *MAPK3*, *MED12*, *MYD88*, *NF1*, *NOTCH1*, *NPM1*, *NRAS*, *PHF6*, *PIK3CD*, *PIK3CG*, *PLCG2*, *PLEKHG5*, *PRKCB*, *PRKD3*, *PTEN*, *PTPN11*, *RAD21*, *RAF1*, *RUNX1*, *SAMHD1*, *SETBP1*, *SF1*, *SF3A1*, *SF3B1*, *SMARCA2*, *SMC1A*, *SMC3*, *SRSF2*, *STAG2*, *SYK*, *TET2*, *TGM7*, *TP53*, *TP73*, *TYK2*, *U2AF1*, *U2AF2*, *WT1*, *XPO1*, *ZMYM3*, *ZRSR2*) was determined by targeted amplicon sequencing using two different gene panels on the MiSeq platform (Illumina, San Diego, CA).^13^ DNA library preparations were performed according to the manufacturer’s instructions. Briefly, samples were pooled and run on the MiSeq machine using the Illumina MiSeq Reagent Kit v3. Sequenced reads were aligned to the hg19 genome build using the Illumina Isis Banded Smith-Waterman aligner. Single nucleotide variant and indel calling were performed using MuTect and VarScan, respectively.^14-15^ The MuCor algorithm was used as the baseline for integrative mutation assessment.^16^ We only considered non-synonymous variants not listed in either the 1000 Genome database or dbSNP142-common variants. All called variants underwent visual inspection of the aligned reads using the Integrative Genomics Viewer (Broad Institute).^17^ All variants that occurred with VAFs of <0.10 were considered wild-type. In addition, variants were excluded when they occurred only in 1 read direction if sequenced in both directions, if the region contained many variants with low quality control cores, or if they occurred in all analyzed samples including run controls. In addition, samples with high background noise were entirely excluded from analysis. Samples were considered non-evaluable for a specific gene if ≥85% of the amplicons covering the target regions within the coding sequence of the gene were sequenced to a depth of <15 reads.

**Supplementary references**

1. Baer MR, George SL, Caligiuri MA, et al. Low-dose interleukin-2 immunotherapy does not improve outcome of patients age 60 years and older with acute myeloid leukemia in first complete remission: Cancer and Leukemia Group B study 9720. J Clin Oncol. 2008;26(30):4934-9.
2. Baer MR, George SL, Sanford BL, et al. Escalation of daunorubicin and addition of etoposidein the ADE regimen in acute myeloid leukemia patients aged 60 years and older: Cancer and Leukemia Group B Study 9720. Leukemia. 2011;25(5):800-7.
3. Marcucci G, Moser B, Blum W, et al. A phase III randomized trial of intensive induction and consolidation chemotherapy ± oblimersen, a pro-apoptotic Bcl-2 antisense oligonucleotide in untreated acute myeloid leukemia patients >60 years old. J Clin Oncol. 2007;25:360s (abstract 7012).
4. Mayer RJ, Davis RB, Schiffer CA, et al. Intensive postremission chemotherapy in adults with acute myeloid leukemia. N Engl J Med. 1994;331(14):896-903.
5. Stone RM, Berg DT, George SL, et al. Granulocyte-macrophage colony-stimulating factor after initial chemotherapy for elderly patients with primary acute myelogenous leukemia. Cancer and Leukemia Group B. N Engl J Med. 1995;332(25):1671-7.
6. Farag SS, George SL, Lee EJ, et al. Postremission therapy with low-dose interleukin 2 with or without intermediate pulse dose interleukin 2 therapy is well tolerated in elderly patients with acute myeloid leukemia: Cancer and Leukemia Group B study 9420. Clin Cancer Res. 2002;8(9):2812-9.
7. Attar EC, Johnson JL, Amrein PC, et al. Bortezomib added to daunorubicin and cytarabine during induction therapy and to intermediate-dose cytarabine for consolidation in patients with previously untreated acute myeloid leukemia age 60 to 75 years: CALGB (Alliance) study 10502. J Clin Oncol. 2013;31(7):923-9.
8. Yin J, LePlant B, Uy GL, et al. Evaluation of event-free survival as a robust end point in untreated acute myeloid leukemia (Alliance A151614). Blood Adv. 2019;3(11):1714-21.
9. Uy GL, Mandrekar SJ, Laumann K, et al. A phase 2 study incorporating sorafenib into the chemotherapy for older adults with *FLT3*-mutated acute myeloid leukemia: CALGB 11001. Blood Adv. 2017;1(5):331-40.
10. Schiffer CA. Intensive post remission therapy of acute myeloid leukemia (AML) with cytoxan/etoposide (CY/VP16) and diazaquone/mitoxantrone (AZQ/MITO). Blood. 1991;78(suppl):460 (abstract 1829).

12. Cheson BD, Cassileth PA, Head DR, et al. Report of the National Cancer Institute-sponsored workshop on definitions of diagnosis and response in acute myeloid leukemia. *J Clin Oncol*. 1990;8(5):813-9.

13. Eisfeld AK, Mrόzek K, Kohlschmidt, et al. The mutational oncoprint of recurrent cytogenetic abnormalities in adult patients with *de novo* acute myeloid leukemia. Leukemia. 2017;31(10):2211-8.

14. Cibulskis K, Lawrence MS, Carter, SL, et al. Sensitive detection of somatic point mutations in impure and heterogeneous cancer samples. Nat Biotechnol. 2013;31(3):213-9.

15. DePristo MA, Banks E, Poplin R, et al. A framework for variation discovery and genotyping using next-generation DNA sequencing data. Nat Genet. 2011;43(5):491-8.

16. Kroll KW, Eisfeld A-K, Lozanski, et al. MuCor: mutation aggregation and correlation. Bioinformatics. 2016;32(10):1557-8.

17. Robinson JT, Thorvaldsdόttir H, Winckler W, et al. Integrative genomics viewer. Nat Biotechnol. 2011;29(1):24-6.

18. Ley TJ, Miller C, Ding L, et al. Genomic and epigenomic landscapes of adult de novo myeloid leukemia. N Engl J Med. 2013;36(22)8:2059-74.

**Supplementary Table S1.** Comparison of frequencies of gene mutations arranged in functional groups (19) of older patients with acute myeloid leukemia assigned to the genetic groups

| **Functional Groups** | ***NPM1*m/** ***FLT3-*ITD‒**  **n=107** | ***KMT2A***  **n=13** | ***IDH2*m**  **n=59** | ***IDH1*m**  **n=35** | ***TP53*m**  **n=50** | **Complex**  **karyotype/ *TP53*wt**  **n=28** | ***FLT3*m**  **n=99** | ***TET2*m or *WT1*m**  **n=42** | **Marker-negative**  **n=56** |
| --- | --- | --- | --- | --- | --- | --- | --- | --- | --- |
| Chromatin Remodeling, n (%) |  |  |  |  |  |  |  |  |  |
| Mutated | 9 (8) | 2 (15) | 17 (29) | 11 (31) | 8 (16) | 5 (18) | 18 (18) | 21 (50) | 26 (46) |
| Wild-type | 98 (92) | 11 (85) | 42 (71) | 24 (69) | 42 (84) | 23 (82) | 81 (82) | 21 (50) | 30 (54) |
| Cohesin Complex, n (%) |  |  |  |  |  |  |  |  |  |
| Mutated | 15 (14) | 0 (0) | 8 (14) | 3 (9) | 2 (4) | 2 (7) | 12 (12) | 8 (19) | 3 (5) |
| Wild-type | 92 (86) | 13 (100) | 51 (86) | 32 (91) | 48 (96) | 26 (93) | 87 (88) | 34 (81) | 53 (95) |
| Kinases, n (%) |  |  |  |  |  |  |  |  |  |
| Mutated | 14 (13) | 2 (15) | 14 (25) | 16 (46) | 3 (6) | 5 (18) | 99 (100) | 3 (7) | 3 (6) |
| Wild-type | 92 (87) | 11 (85) | 42 (75) | 19 (54) | 47 (94) | 23 (82) | 0 (0) | 38 (93) | 50 (94) |
| Methylation-related, n (%) |  |  |  |  |  |  |  |  |  |
| Mutated | 93 (87) | 3 (23) | 59 (100) | 35 (100) | 12 (24) | 9 (32) | 58 (59) | 39 (93) | 6 (11) |
| Wild-type | 14 (13) | 10 (77) | 0 (0) | 0 (0) | 38 (76) | 19 (68) | 41 (41) | 3 (7) | 50 (89) |
| *NPM1*, n (%) |  |  |  |  |  |  |  |  |  |
| Mutated | 107 (100) | 0 (0) | 8 (14) | 6 (17) | 1 (2) | 1 (4) | 54 (55) | 0 (0) | 0 (0) |
| Wild-type | 0 (0) | 13 (100) | 51 (86) | 29 (83) | 49 (98) | 27 (96) | 45 (45) | 42 (100) | 56 (100) |
| RAS Pathway, n (%) |  |  |  |  |  |  |  |  |  |
| Mutated | 50 (47) | 2 (15) | 8 (14) | 2 (6) | 1 (2) | 4 (14) | 9 (9) | 10 (24) | 20 (36) |
| Wild-type | 57 (53) | 11 (85) | 51 (86) | 33 (94) | 49 (98) | 24 (86) | 90 (91) | 32 (76) | 36 (64) |
| Spliceosome, n (%) |  |  |  |  |  |  |  |  |  |
| Mutated | 30 (28) | 2 (15) | 33 (57) | 12 (34) | 7 (14) | 10 (36) | 26 (26) | 25 (61) | 25 (45) |
| Wild-type | 77 (72) | 11 (85) | 25 (43) | 23 (66) | 43 (86) | 18 (64) | 73 (74) | 16 (39) | 31 (55) |
| Transcription Factors, n (%) |  |  |  |  |  |  |  |  |  |
| Mutated | 7 (7) | 1 (9) | 16 (29) | 7 (21) | 7 (15) | 3 (13) | 28 (29) | 14 (36) | 32 (64) |
| Wild-type | 98 (93) | 10 (91) | 40 (71) | 26 (79) | 40 (85) | 21 (88) | 68 (71) | 25 (64) | 18 (36) |
| Tumor Suppressors, n (%) |  |  |  |  |  |  |  |  |  |
| Mutated | 6 (6) | 2 (15) | 5 (8) | 5 (14) | 50 (100) | 3 (11) | 15 (15) | 5 (12) | 3 (5) |
| Wild-type | 101 (94) | 11 (85) | 54 (92) | 30 (86) | 0 (0) | 25 (89) | 84 (85) | 37 (88) | 53 (95) |

Abbreviations: *m*, mutated; *n*, number.

^a^ A given functional group is considered mutated if at least one gene belonging to this group is mutated. Functional groups comprise specific genes as follows: chromatin remodeling: *ASXL1*, *BCOR*, *BCORL1*, *EZH2* and *SMARCA2*; cohesin complex: *RAD21*, *SMC1A*, *SMC3* and *STAG2*; kinases: *AXL*, *FLT3*-ITD, *FLT3*-TKD, *KIT* and *TYK2*; methylation-related: *DNMT3A*, *IDH1*, *IDH2* and *TET2*; NPM1: *NPM1*; RAS pathway: *CBL*, *KRAS*, *NRAS* and *PTPN11*; spliceosome: *SF3B1*, *SRSF2*, *U2AF1* and *ZRSR2*; transcription factors: *CEBPA* biallelic, *ETV6*, *GATA2*, *IKZF1*, *NOTCH1* and *RUNX1*; and tumor suppressors: *PHF6*, *TP53* and *WT1* (19).

**Supplementary Table S2.** Comparison of frequencies of single gene mutations detected in older patients with acute myeloid leukemia assigned to the genetic groups

| **Gene** | ***NPM1*m/** ***FLT3-*ITD‒**  **n=107** | ***KMT2A***  **n=13** | ***IDH2*m**  **n=59** | ***IDH1*m**  **n=35** | ***TP53*m**  **n=50** | **Complex**  **karyotype/ *TP53*wt**  **n=28** | ***FLT3*m**  **n=99** | ***TET2*m or *WT1*m**  **n=42** | **Marker-**  **negative**  **n=56** |
| --- | --- | --- | --- | --- | --- | --- | --- | --- | --- |
| *AKT1*, n (%) |  |  |  |  |  |  |  |  |  |
| Mutated | 0 (0) | 0 (0) | 0 (0) | 0 (0) | 0 (0) | 0 (0) | 1 (1) | 0 (0) | 0 (0) |
| Wild-type | 107 (100) | 13 (100) | 59 (100) | 35 (100) | 50 (100) | 28 (100) | 98 (99) | 42 (100) | 56 (100) |
| *ARAF*, n (%) |  |  |  |  |  |  |  |  |  |
| Mutated | 0 (0) | 0 (0) | 0 (0) | 0 (0) | 0 (0) | 0 (0) | 0 (0) | 1 (2) | 1 (2) |
| Wild-type | 107 (100) | 13 (100) | 59 (100) | 35 (100) | 50 (100) | 28 (100) | 99 (100) | 41 (98) | 55 (98) |
| *ASXL1*, n (%) |  |  |  |  |  |  |  |  |  |
| Mutated | 2 (2) | 1 (8) | 9 (15) | 6 (17) | 4 (8) | 4 (14) | 7 (7) | 13 (31) | 14 (25) |
| Wild-type | 105 (98) | 12 (92) | 50 (85) | 29 (83) | 46 (92) | 24 (86) | 92 (93) | 29 (69) | 42 (75) |
| *ATM*, n (%) |  |  |  |  |  |  |  |  |  |
| Mutated | 0 (0) | 0 (0) | 0 (0) | 0 (0) | 0 (0) | 0 (0) | 0 (0) | 1 (2) | 2 (4) |
| Wild-type | 107 (100) | 13 (100) | 59 (100) | 35 (100) | 50 (100) | 28 (100) | 99 (100) | 41 (98) | 54 (96) |
| *AXL*, n (%) |  |  |  |  |  |  |  |  |  |
| Mutated | 0 (0) | 0 (0) | 0 (0) | 0 (0) | 0 (0) | 1 (4) | 0 (0) | 0 (0) | 0 (0) |
| Wild-type | 107 (100) | 13 (100) | 59 (100) | 35 (100) | 50 (100) | 27 (96) | 99 (100) | 42 (100) | 56 (100) |
| *BCOR*, n (%) |  |  |  |  |  |  |  |  |  |
| Mutated | 4 (4) | 0 (0) | 8 (14) | 4 (11) | 1 (2) | 1 (4) | 3 (3) | 5 (12) | 12 (21) |
| Wild-type | 103 (96) | 13 (100) | 51 (86) | 31 (89) | 49 (98) | 27 (96) | 96 (97) | 37 (88) | 44 (79) |
| *BCORL1*, n (%) |  |  |  |  |  |  |  |  |  |
| Mutated | 1 (1) | 0 (0) | 2 (3) | 1 (3) | 0 (0) | 0 (0) | 4 (4) | 4 (10) | 5 (9) |
| Wild-type | 106 (99) | 13 (100) | 57 (97) | 34 (97) | 50 (100) | 28 (100) | 95 (96) | 38 (90) | 51 (91) |
| *BRAF*, n (%) |  |  |  |  |  |  |  |  |  |
| Mutated | 1 (1) | 0 (0) | 0 (0) | 0 (0) | 0 (0) | 0 (0) | 0 (0) | 2 (5) | 0 (0) |
| Wild-type | 106 (99) | 13 (100) | 59 (100) | 35 (100) | 50 (100) | 28 (100) | 99 (100) | 40 (95) | 56 (100) |
| *BRD4*, n (%) |  |  |  |  |  |  |  |  |  |
| Mutated | 2 (2) | 0 (0) | 0 (0) | 0 (0) | 0 (0) | 0 (0) | 0 (0) | 0 (0) | 1 (2) |
| Wild-type | 84 (98) | 10 (100) | 51 (100) | 27 (100) | 31 (100) | 23 (100) | 76 (100) | 35 (100) | 44 (98) |
| *BRINP3*, n (%) |  |  |  |  |  |  |  |  |  |
| Mutated | 1 (1) | 0 (0) | 2 (3) | 0 (0) | 2 (4) | 0 (0) | 0 (0) | 0 (0) | 4 (7) |
| Wild-type | 106 (99) | 13 (100) | 57 (97) | 35 (100) | 48 (96) | 28 (100) | 99 (100) | 42 (100) | 52 (93) |
| *BTK*, n (%) |  |  |  |  |  |  |  |  |  |
| Mutated | 0 (0) | 0 (0) | 0 (0) | 0 (0) | 0 (0) | 0 (0) | 1 (1) | 0 (0) | 0 (0) |
| Wild-type | 107 (100) | 13 (100) | 59 (100) | 35 (100) | 50 (100) | 28 (100) | 98 (99) | 42 (100) | 56 (100) |
| *CBL*, n (%) |  |  |  |  |  |  |  |  |  |
| Mutated | 5 (5) | 0 (0) | 0 (0) | 1 (3) | 0 (0) | 0 (0) | 2 (2) | 5 (12) | 2 (4) |
| Wild-type | 102 (95) | 13 (100) | 59 (100) | 34 (97) | 50 (100) | 28 (100) | 97 (98) | 37 (88) | 54 (96) |
| *CCND1*, n (%) |  |  |  |  |  |  |  |  |  |
| Mutated | 1 (1) | 0 (0) | 1 (2) | 0 (0) | 0 (0) | 0 (0) | 0 (0) | 0 (0) | 0 (0) |
| Wild-type | 106 (99) | 13 (100) | 58 (98) | 35 (100) | 50 (100) | 28 (100) | 99 (100) | 42 (100) | 56 (100) |
| *CCND2*, n (%) |  |  |  |  |  |  |  |  |  |
| Mutated | 0 (0) | 0 (0) | 0 (0) | 0 (0) | 0 (0) | 0 (0) | 1 (1) | 0 (0) | 0 (0) |
| Wild-type | 107 (100) | 13 (100) | 59 (100) | 35 (100) | 50 (100) | 28 (100) | 98 (99) | 42 (100) | 56 (100) |
| *CEBPA*, n (%) |  |  |  |  |  |  |  |  |  |
| Mutated | 2 (2) | 0 (0) | 1 (2) | 1 (3) | 0 (0) | 0 (0) | 2 (2) | 5 (14) | 4 (8) |
| Wild-type | 103 (98) | 11 (100) | 55 (98) | 32 (97) | 47 (100) | 24 (100) | 93 (98) | 32 (86) | 45 (92) |
| *CSNK1A1*, n (%) |  |  |  |  |  |  |  |  |  |
| Mutated | 0 (0) | 0 (0) | 0 (0) | 0 (0) | 0 (0) | 0 (0) | 1 (1) | 0 (0) | 0 (0) |
| Wild-type | 107 (100) | 13 (100) | 59 (100) | 35 (100) | 50 (100) | 28 (100) | 98 (99) | 42 (100) | 56 (100) |
| *CTNNB1*, n (%) |  |  |  |  |  |  |  |  |  |
| Mutated | 0 (0) | 0 (0) | 0 (0) | 0 (0) | 0 (0) | 0 (0) | 0 (0) | 1 (2) | 0 (0) |
| Wild-type | 107 (100) | 13 (100) | 59 (100) | 35 (100) | 50 (100) | 28 (100) | 99 (100) | 41 (98) | 56 (100) |
| *DNMT3A*, n (%) |  |  |  |  |  |  |  |  |  |
| Mutated  R882  Non-R882 | 37 (35)  19  18 | 2 (15)  0  2 | 18 (31)  7  11 | 14 (40)  10  4 | 5 (10)  1  4 | 1 (4)  0  1 | 40 (40)  29  12 | 9 (21)  7  2 | 4 (7)  4  0 |
| Wild-type | 70 (65) | 11 (85) | 41 (69) | 21 (60) | 45 (90) | 27 (96) | 59 (60) | 33 (79) | 52 (93) |
| *ETV6*, n (%) |  |  |  |  |  |  |  |  |  |
| Mutated | 1 (1) | 0 (0) | 2 (3) | 0 (0) | 2 (4) | 0 (0) | 3 (3) | 2 (5) | 3 (5) |
| Wild-type | 106 (99) | 13 (100) | 57 (97) | 35 (100) | 48 (96) | 28 (100) | 96 (97) | 40 (95) | 53 (95) |
| *EZH2*, n (%) |  |  |  |  |  |  |  |  |  |
| Mutated | 1 (1) | 0 (0) | 0 (0) | 2 (6) | 1 (2) | 0 (0) | 3 (3) | 5 (12) | 6 (11) |
| Wild-type | 106 (99) | 13 (100) | 59 (100) | 33 (94) | 49 (98) | 28 (100) | 96 (97) | 37 (88) | 50 (89) |
| *FBXW7*, n (%) |  |  |  |  |  |  |  |  |  |
| Mutated | 0 (0) | 0 (0) | 0 (0) | 0 (0) | 1 (2) | 0 (0) | 0 (0) | 0 (0) | 0 (0) |
| Wild-type | 107 (100) | 13 (100) | 59 (100) | 35 (100) | 49 (98) | 27 (100) | 99 (100) | 42 (100) | 56 (100) |
| *FLT3*-ITD, n (%) |  |  |  |  |  |  |  |  |  |
| Present | 0 (0) | 2 (15) | 13 (22) | 12 (34) | 2 (4) | 2 (7) | 93 (94) | 0 (0) | 0 (0) |
| Absent | 107 (100) | 11 (85) | 46 (78) | 23 (66) | 48 (96) | 26 (93) | 6 (6) | 42 (100) | 56 (100) |
| *FLT3*-TKD, n (%) |  |  |  |  |  |  |  |  |  |
| Present | 14 (13) | 0 (0) | 2 (3) | 5 (14) | 0 (0) | 2 (7) | 8 (8) | 1 (2) | 0 (0) |
| Absent | 93 (87) | 12 (100) | 57 (97) | 30 (86) | 50 (100) | 25 (93) | 90 (92) | 41 (98) | 56 (100) |
| *GATA1*, n (%) |  |  |  |  |  |  |  |  |  |
| Mutated | 0 (0) | 0 (0) | 0 (0) | 0 (0) | 0 (0) | 0 (0) | 1 (1) | 0 (0) | 0 (0) |
| Wild-type | 86 (100) | 10 (100) | 48 (100) | 27 (100) | 29 (100) | 23 (100) | 75 (99) | 35 (100) | 45 (100) |
| *GATA2*, n (%) |  |  |  |  |  |  |  |  |  |
| Mutated | 1 (1) | 0 (0) | 0 (0) | 0 (0) | 0 (0) | 0 (0) | 1 (1) | 1 (2) | 5 (9) |
| Wild-type | 106 (99) | 13 (100) | 59 (100) | 35 (100) | 50 (100) | 28 (100) | 98 (99) | 41 (98) | 51 (91) |
| *GSK3B*, n (%) |  |  |  |  |  |  |  |  |  |
| Mutated | 0 (0) | 0 (0) | 0 (0) | 0 (0) | 0 (0) | 0 (0) | 0 (0) | 1 (2) | 0 (0) |
| Wild-type | 107 (100) | 13 (100) | 59 (100) | 35 (100) | 50 (100) | 28 (100) | 99 (100) | 41 (98) | 56 (100) |
| *HIST1H1E*, n (%) |  |  |  |  |  |  |  |  |  |
| Mutated | 4 (4) | 0 (0) | 0 (0) | 0 (0) | 3 (6) | 0 (0) | 1 (1) | 1 (2) | 1 (2) |
| Wild-type | 103 (96) | 13 (100) | 59 (100) | 35 (100) | 47 (94) | 28 (100) | 98 (99) | 41 (98) | 55 (98) |
| *HNRNPK*, n (%) |  |  |  |  |  |  |  |  |  |
| Mutated | 2 (2) | 0 (0) | 0 (0) | 0 (0) | 0 (0) | 0 (0) | 0 (0) | 0 (0) | 1 (2) |
| Wild-type | 105 (98) | 13 (100) | 59 (100) | 35 (100) | 50 (100) | 28 (100) | 99 (100) | 42 (100) | 55 (98) |
| *IDH1*, n (%) |  |  |  |  |  |  |  |  |  |
| Mutated | 22 (21) | 0 (0) | 0 (0) | 35 (100) | 0 (0) | 0 (0) | 0 (0) | 0 (0) | 0 (0) |
| Wild-type | 85 (79) | 13 (100) | 59 (100) | 0 (0) | 50 (100) | 28 (100) | 99 (100) | 42 (100) | 56 (100) |
| *IDH2*, n (%) |  |  |  |  |  |  |  |  |  |
| Mutated | 22 (21) | 0 (0) | 59 (100) | 0 (0) | 0 (0) | 0 (0) | 2 (2) | 0 (0) | 3 (5) |
| Wild-type | 85 (79) | 13 (100) | 0 (0) | 35 (100) | 50 (100) | 28 (100) | 97 (98) | 42 (100) | 53 (95) |
| *IKZF1*, n (%) |  |  |  |  |  |  |  |  |  |
| Mutated | 0 (0) | 0 (0) | 2 (3) | 0 (0) | 1 (2) | 1 (4) | 3 (3) | 0 (0) | 2 (4) |
| Wild-type | 107 (100) | 13 (100) | 57 (97) | 35 (100) | 49 (98) | 27 (96) | 96 (97) | 42 (100) | 54 (96) |
| *IKZF3*, n (%) |  |  |  |  |  |  |  |  |  |
| Mutated | 0 (0) | 1 (8) | 0 (0) | 0 (0) | 0 (0) | 0 (0) | 0 (0) | 0 (0) | 0 (0) |
| Wild-type | 107 (100) | 12 (92) | 59 (100) | 35 (100) | 50 (100) | 28 (100) | 99 (100) | 42 (100) | 56 (100) |
| *JAK1*, n (%) |  |  |  |  |  |  |  |  |  |
| Mutated | 0 (0) | 0 (0) | 0 (0) | 0 (0) | 0 (0) | 1 (4) | 1 (1) | 0 (0) | 1 (2) |
| Wild-type | 107 (100) | 13 (100) | 59 (100) | 35 (100) | 50 (100) | 27 (96) | 98 (99) | 42 (100) | 55 (98) |
| *JAK2*, n (%) |  |  |  |  |  |  |  |  |  |
| Mutated | 0 (0) | 0 (0) | 1 (2) | 0 (0) | 0 (0) | 0 (0) | 1 (1) | 0 (0) | 0 (0) |
| Wild-type | 107 (100) | 12 (100) | 55 (98) | 35 (100) | 50 (100) | 27 (100) | 96 (99) | 42 (100) | 55 (100) |
| *JAK3*, n (%) |  |  |  |  |  |  |  |  |  |
| Mutated | 0 (0) | 1 (8) | 0 (0) | 0 (0) | 0 (0) | 1 (4) | 1 (1) | 2 (5) | 0 (0) |
| Wild-type | 107 (100) | 12 (92) | 59 (100) | 35 (100) | 50 (100) | 27 (96) | 98 (99) | 40 (95) | 56 (100) |
| *KIT*, n (%) |  |  |  |  |  |  |  |  |  |
| Mutated | 0 (0) | 0 (0) | 0 (0) | 0 (0) | 1 (2) | 0 (0) | 3 (3) | 1 (2) | 1 (2) |
| Wild-type | 107 (100) | 13 (100) | 57 (100) | 35 (100) | 49 (98) | 28 (100) | 96 (97) | 41 (98) | 55 (98) |
| *KLHL6*, n (%) |  |  |  |  |  |  |  |  |  |
| Mutated | 0 (0) | 0 (0) | 0 (0) | 0 (0) | 0 (0) | 1 (4) | 0 (0) | 0 (0) | 0 (0) |
| Wild-type | 107 (100) | 13 (100) | 59 (100) | 35 (100) | 50 (100) | 27 (96) | 99 (100) | 42 (100) | 56 (100) |
| *KMT2A*, n (%) |  |  |  |  |  |  |  |  |  |
| Mutated | 0 (0) | 0 (0) | 0 (0) | 0 (0) | 0 (0) | 1 (4) | 0 (0) | 0 (0) | 1 (2) |
| Wild-type | 107 (100) | 13 (100) | 59 (100) | 35 (100) | 50 (100) | 27 (96) | 99 (100) | 42 (100) | 55 (98) |
| *KRAS*, n (%) |  |  |  |  |  |  |  |  |  |
| Mutated | 6 (6) | 1 (8) | 1 (2) | 0 (0) | 0 (0) | 0 (0) | 0 (0) | 1 (2) | 5 (9) |
| Wild-type | 101 (94) | 12 (92) | 58 (98) | 35 (100) | 50 (100) | 28 (100) | 99 (100) | 41 (98) | 51 (91) |
| *MAPK3*, n (%) |  |  |  |  |  |  |  |  |  |
| Mutated | 0 (0) | 0 (0) | 0 (0) | 0 (0) | 1 (2) | 0 (0) | 0 (0) | 0 (0) | 0 (0) |
| Wild-type | 107 (100) | 13 (100) | 59 (100) | 35 (100) | 49 (98) | 28 (100) | 99 (100) | 42 (100) | 56 (100) |
| *MED12*, n (%) |  |  |  |  |  |  |  |  |  |
| Mutated | 1 (1) | 0 (0) | 2 (3) | 1 (3) | 0 (0) | 2 (7) | 1 (1) | 3 (7) | 1 (2) |
| Wild-type | 106 (99) | 13 (100) | 57 (97) | 34 (97) | 50 (100) | 26 (93) | 98 (99) | 39 (93) | 55 (98) |
| *MYD88*, n (%) |  |  |  |  |  |  |  |  |  |
| Mutated | 0 (0) | 0 (0) | 0 (0) | 1 (3) | 0 (0) | 0 (0) | 0 (0) | 0 (0) | 0 (0) |
| Wild-type | 107 (100) | 13 (100) | 59 (100) | 34 (97) | 50 (100) | 28 (100) | 99 (100) | 42 (100) | 56 (100) |
| *NOTCH1*, n (%) |  |  |  |  |  |  |  |  |  |
| Mutated | 1 (1) | 0 (0) | 2 (4) | 1 (4) | 1 (3) | 0 (0) | 2 (3) | 0 (0) | 1 (2) |
| Wild-type | 86 (99) | 10 (100) | 46 (96) | 26 (96) | 28 (97) | 23 (100) | 74 (97) | 35 (100) | 44 (98) |
| *NPM1*, n (%) |  |  |  |  |  |  |  |  |  |
| Mutated | 107 (100) | 0 (0) | 8 (14) | 6 (17) | 1 (2) | 1 (4) | 54 (55) | 0 (0) | 0 (0) |
| Wild-type | 0 (0) | 13 (100) | 51 (86) | 29 (83) | 49 (98) | 27 (96) | 45 (45) | 42 (100) | 56 (100) |
| *NRAS*, n (%) |  |  |  |  |  |  |  |  |  |
| Mutated | 22 (21) | 1 (8) | 7 (12) | 0 (0) | 1 (2) | 3 (11) | 4 (4) | 3 (7) | 12 (21) |
| Wild-type | 85 (79) | 12 (92) | 52 (88) | 35 (100) | 49 (98) | 25 (89) | 95 (96) | 39 (93) | 44 (79) |
| *PHF6*, n (%) |  |  |  |  |  |  |  |  |  |
| Mutated | 4 (4) | 0 (0) | 2 (3) | 2 (6) | 1 (2) | 1 (4) | 3 (3) | 1 (2) | 2 (4) |
| Wild-type | 103 (96) | 13 (100) | 57 (97) | 33 (94) | 49 (98) | 27 (96) | 96 (97) | 41 (98) | 54 (96) |
| *PIK3CD*, n (%) |  |  |  |  |  |  |  |  |  |
| Mutated | 1 (1) | 0 (0) | 0 (0) | 1 (3) | 0 (0) | 0 (0) | 0 (0) | 0 (0) | 1 (2) |
| Wild-type | 101 (99) | 13 (100) | 57 (100) | 31 (97) | 46 (100) | 26 (100) | 89 (100) | 36 (100) | 51 (98) |
| *PIK3CG*, n (%) |  |  |  |  |  |  |  |  |  |
| Mutated | 2 (2) | 0 (0) | 1 (2) | 1 (3) | 0 (0) | 0 (0) | 3 (3) | 0 (0) | 0 (0) |
| Wild-type | 105 (98) | 13 (100) | 58 (98) | 34 (97) | 50 (100) | 28 (100) | 96 (97) | 42 (100) | 56 (100) |
| *PLCG2*, n (%) |  |  |  |  |  |  |  |  |  |
| Mutated | 0 (0) | 0 (0) | 0 (0) | 0 (0) | 1 (2) | 0 (0) | 1 (1) | 1 (2) | 1 (2) |
| Wild-type | 107 (100) | 13 (100) | 59 (100) | 35 (100) | 49 (98) | 28 (100) | 98 (99) | 41 (98) | 55 (98) |
| *PLEKHG5*, n (%) |  |  |  |  |  |  |  |  |  |
| Mutated | 0 (0) | 0 (0) | 0 (0) | 0 (0) | 0 (0) | 0 (0) | 0 (0) | 1 (2) | 0 (0) |
| Wild-type | 106 (100) | 13 (100) | 58 (100) | 35 (100) | 49 (100) | 28 (100) | 98 (100) | 40 (98) | 56 (100) |
| *PRKCB*, n (%) |  |  |  |  |  |  |  |  |  |
| Mutated | 2 (2) | 0 (0) | 0 (0) | 1 (3) | 2 (4) | 0 (0) | 0 (0) | 0 (0) | 1 (2) |
| Wild-type | 105 (98) | 13 (100) | 59 (100) | 34 (97) | 48 (96) | 28 (100) | 99 (100) | 42 (100) | 55 (98) |
| *PRKD3*, n (%) |  |  |  |  |  |  |  |  |  |
| Mutated | 1 (1) | 1 (8) | 1 (2) | 0 (0) | 0 (0) | 0 (0) | 0 (0) | 0 (0) | 0 (0) |
| Wild-type | 106 (99) | 12 (92) | 58 (98) | 35 (100) | 50 (100) | 28 (100) | 99 (100) | 42 (100) | 56 (100) |
| *PTPN11*, n (%) |  |  |  |  |  |  |  |  |  |
| Mutated | 17 (16) | 0 (0) | 1 (2) | 1 (3) | 0 (0) | 1 (4) | 3 (3) | 1 (2) | 3 (5) |
| Wild-type | 90 (84) | 13 (100) | 58 (98) | 34 (97) | 50 (100) | 27 (96) | 96 (97) | 41 (98) | 53 (95) |
| *RAD21*, n (%) |  |  |  |  |  |  |  |  |  |
| Mutated | 2 (2) | 0 (0) | 0 (0) | 1 (3) | 0 (0) | 0 (0) | 3 (3) | 0 (0) | 0 (0) |
| Wild-type | 105 (98) | 13 (100) | 59 (100) | 34 (97) | 50 (100) | 28 (100) | 96 (97) | 42 (100) | 56 (100) |
| *RAF1*, n (%) |  |  |  |  |  |  |  |  |  |
| Mutated | 1 (1) | 0 (0) | 0 (0) | 1 (3) | 1 (2) | 0 (0) | 1 (1) | 0 (0) | 0 (0) |
| Wild-type | 106 (99) | 13 (100) | 59 (100) | 34 (97) | 49 (98) | 28 (100) | 98 (99) | 42 (100) | 56 (100) |
| *RUNX1*, n (%) |  |  |  |  |  |  |  |  |  |
| Mutated | 2 (2) | 1 (8) | 11 (19) | 5 (14) | 3 (6) | 2 (7) | 19 (19) | 9 (21) | 24 (43) |
| Wild-type | 105 (98) | 12 (92) | 48 (81) | 30 (86) | 47 (94) | 26 (93) | 80 (81) | 33 (79) | 32 (57) |
| *SAMHD1*, n (%) |  |  |  |  |  |  |  |  |  |
| Mutated | 0 (0) | 0 (0) | 1 (2) | 0 (0) | 2 (4) | 0 (0) | 1 (1) | 0 (0) | 0 (0) |
| Wild-type | 107 (100) | 13 (100) | 58 (98) | 35 (100) | 48 (96) | 28 (100) | 98 (99) | 42 (100) | 56 (100) |
| *SETBP1*, n (%) |  |  |  |  |  |  |  |  |  |
| Mutated | 2 (2) | 0 (0) | 0 (0) | 0 (0) | 1 (2) | 0 (0) | 2 (2) | 0 (0) | 2 (4) |
| Wild-type | 105 (98) | 13 (100) | 59 (100) | 35 (100) | 49 (98) | 28 (100) | 97 (98) | 42 (100) | 54 (96) |
| *SF1*, n (%) |  |  |  |  |  |  |  |  |  |
| Mutated | 2 (2) | 2 (15) | 0 (0) | 2 (6) | 1 (2) | 0 (0) | 1 (1) | 0 (0) | 1 (2) |
| Wild-type | 105 (98) | 11 (85) | 59 (100) | 33 (94) | 49 (98) | 28 (100) | 98 (99) | 42 (100) | 55 (98) |
| *SF3A1*, n (%) |  |  |  |  |  |  |  |  |  |
| Mutated | 2 (2) | 0 (0) | 1 (2) | 1 (3) | 0 (0) | 0 (0) | 0 (0) | 1 (2) | 1 (2) |
| Wild-type | 105 (98) | 13 (100) | 58 (98) | 34 (97) | 50 (100) | 28 (100) | 99 (100) | 41 (98) | 55 (98) |
| *SF3B1*, n (%) |  |  |  |  |  |  |  |  |  |
| Mutated | 5 (5) | 0 (0) | 0 (0) | 0 (0) | 1 (2) | 1 (4) | 4 (4) | 3 (7) | 5 (9) |
| Wild-type | 102 (95) | 13 (100) | 59 (100) | 35 (100) | 49 (98) | 27 (96) | 95 (96) | 39 (93) | 51 (91) |
| *SMARCA2*, n (%) |  |  |  |  |  |  |  |  |  |
| Mutated | 1 (1) | 1 (8) | 0 (0) | 0 (0) | 2 (4) | 0 (0) | 1 (1) | 3 (7) | 0 (0) |
| Wild-type | 106 (99) | 12 (92) | 59 (100) | 35 (100) | 48 (96) | 28 (100) | 98 (99) | 39 (93) | 56 (100) |
| *SMC1A*, n (%) |  |  |  |  |  |  |  |  |  |
| Mutated | 5 (5) | 0 (0) | 4 (7) | 0 (0) | 0 (0) | 1 (4) | 2 (2) | 2 (5) | 2 (4) |
| Wild-type | 102 (95) | 13 (100) | 55 (93) | 35 (100) | 50 (100) | 27 (96) | 97 (98) | 40 (95) | 54 (96) |
| *SMC3*, n (%) |  |  |  |  |  |  |  |  |  |
| Mutated | 3 (3) | 0 (0) | 0 (0) | 0 (0) | 1 (2) | 0 (0) | 4 (4) | 0 (0) | 1 (2) |
| Wild-type | 104 (97) | 13 (100) | 59 (100) | 35 (100) | 49 (98) | 28 (100) | 95 (96) | 42 (100) | 55 (98) |
| *SRSF2*, n (%) |  |  |  |  |  |  |  |  |  |
| Mutated | 19 (18) | 1 (8) | 30 (52) | 8 (23) | 3 (6) | 5 (19) | 6 (6) | 15 (37) | 10 (18) |
| Wild-type | 88 (82) | 12 (92) | 28 (48) | 27 (77) | 47 (94) | 22 (81) | 93 (94) | 26 (63) | 46 (82) |
| *STAG2*, n (%) |  |  |  |  |  |  |  |  |  |
| Mutated | 5 (5) | 0 (0) | 4 (7) | 2 (6) | 1 (2) | 1 (4) | 3 (3) | 6 (14) | 0 (0) |
| Wild-type | 102 (95) | 13 (100) | 55 (93) | 33 (94) | 49 (98) | 27 (96) | 96 (97) | 36 (86) | 56 (100) |
| *SYK*, n (%) |  |  |  |  |  |  |  |  |  |
| Mutated | 2 (2) | 0 (0) | 0 (0) | 1 (3) | 0 (0) | 0 (0) | 2 (2) | 0 (0) | 0 (0) |
| Wild-type | 105 (98) | 13 (100) | 59 (100) | 34 (97) | 50 (100) | 28 (100) | 97 (98) | 42 (100) | 56 (100) |
| *TET2*, n (%) |  |  |  |  |  |  |  |  |  |
| Mutated | 35 (33) | 1 (8) | 5 (8) | 5 (14) | 9 (18) | 9 (32) | 25 (25) | 39 (93) | 0 (0) |
| Wild-type | 72 (67) | 12 (92) | 54 (92) | 30 (86) | 41 (82) | 19 (68) | 74 (75) | 3 (7) | 56 (100) |
| *TGM7*, n (%) |  |  |  |  |  |  |  |  |  |
| Mutated | 0 (0) | 0 (0) | 1 (2) | 0 (0) | 0 (0) | 0 (0) | 0 (0) | 1 (2) | 0 (0) |
| Wild-type | 107 (100) | 13 (100) | 58 (98) | 35 (100) | 49 (100) | 27 (100) | 98 (100) | 41 (98) | 56 (100) |
| *TP53*, n (%) |  |  |  |  |  |  |  |  |  |
| Mutated | 1 (1) | 1 (8) | 3 (5) | 3 (9) | 50 (100) | 2 (7) | 1 (1) | 0 (0) | 1 (2) |
| Wild-type | 106 (99) | 12 (92) | 56 (95) | 32 (91) | 0 (0) | 26 (93) | 98 (99) | 42 (100) | 55 (98) |
| *TYK2*, n (%) |  |  |  |  |  |  |  |  |  |
| Mutated | 0 (0) | 0 (0) | 2 (3) | 0 (0) | 0 (0) | 0 (0) | 2 (2) | 1 (2) | 2 (4) |
| Wild-type | 107 (100) | 13 (100) | 57 (97) | 35 (100) | 50 (100) | 28 (100) | 97 (98) | 41 (98) | 54 (96) |
| *U2AF1*, n (%) |  |  |  |  |  |  |  |  |  |
| Mutated | 3 (3) | 0 (0) | 2 (3) | 4 (11) | 1 (2) | 1 (4) | 12 (12) | 4 (10) | 11 (20) |
| Wild-type | 104 (97) | 13 (100) | 57 (97) | 31 (89) | 49 (98) | 27 (96) | 87 (88) | 38 (90) | 45 (80) |
| *WT1*, n (%) |  |  |  |  |  |  |  |  |  |
| Mutated | 1 (1) | 1 (8) | 0 (0) | 0 (0) | 0 (0) | 0 (0) | 11 (11) | 4 (10) | 0 (0) |
| Wild-type | 106 (99) | 12 (92) | 59 (100) | 35 (100) | 50 (100) | 28 (100) | 88 (89) | 38 (90) | 56 (100) |
| *XPO1*, n (%) |  |  |  |  |  |  |  |  |  |
| Mutated | 2 (2) | 0 (0) | 0 (0) | 0 (0) | 0 (0) | 0 (0) | 0 (0) | 1 (2) | 0 (0) |
| Wild-type | 105 (98) | 13 (100) | 59 (100) | 35 (100) | 50 (100) | 28 (100) | 99 (100) | 41 (98) | 56 (100) |
| *ZRSR2*, n (%) |  |  |  |  |  |  |  |  |  |
| Mutated | 5 (5) | 1 (8) | 2 (3) | 0 (0) | 3 (6) | 3 (11) | 4 (4) | 4 (10) | 2 (4) |
| Wild-type | 102 (95) | 12 (92) | 57 (97) | 35 (100) | 47 (94) | 25 (89) | 95 (96) | 38 (90) | 54 (96) |

Abbreviation: *m,* mutated, *n*, number.

The following genes were included in sequencing but not included in the table because no mutation in these genes was detected in any of the separate genomic groups: *BCL2*, *ILR7*, *MAPK1*, *NF1*, *PTEN*, *TP73*, *U2AF2*, and *ZMYM3*.
